# Measuring bonding or attachment in the parent-infant-relationship: A systematic review of parent-report assessment measures, their psychometric properties and clinical utility

**DOI:** 10.1016/j.cpr.2020.101906

**Published:** 2020-12

**Authors:** A. Wittkowski, S. Vatter, A. Muhinyi, C. Garrett, M. Henderson

**Affiliations:** a Division of Psychology and Mental Health, School of Health Sciences, Faculty of Biology, Medicine and Health, The University of Manchester, Manchester Academic Health Science Centre, Manchester M13 9PL, UK; b Greater Manchester Mental Health NHS Foundation Trust, Department of Clinical Psychology, Laureate House, Wythenshawe Hospital, Southmoor Road, Wythenshawe, Manchester M23 9LT, UK; c MRC/CSO Social and Public Health Sciences Unit, Institute of Health and Wellbeing, University of Glasgow, 200 Renfield Street, Glasgow G2 3AX, UK

**Keywords:** Measurement, Reliability, Validity, Mothers, Fathers, Quality assessment

## Abstract

**Background:**

Meaningful, valid and reliable self-report measures can facilitate the identification of important parent-infant-relationship factors, relevant intervention development and subsequent evaluation in community and clinical contexts. We aimed at identifying all available parent-report measures of the parent-infant-relationship or bond and to appraise their psychometric and clinimetric properties.

**Method:**

A systematic review (PROSPERO: CRD42017078512) was conducted using the, 2018 COSMIN criteria. Eight electronic databases were searched. Papers describing the development of self-report measures of the parent-infant-bond, attachment or relationship from pregnancy until two years postpartum or the assessment of their psychometric properties were included.

**Results:**

Sixty-five articles evaluating 17 original measures and 13 modified versions were identified and reviewed. The studies' methodological quality (risk of bias) varied between ‘very good’ and ‘inadequate’ depending on the measurement property assessed; however, scale development studies were mostly of ‘inadequate’ quality. Although most measures had good clinical utility, the psychometric evaluation of their properties was largely poor. The original or modified versions of the Postpartum Bonding Questionnaire collectively received the strongest psychometric evaluation ratings with high quality of evidence.

**Conclusions:**

This novel review revealed that only a few antenatal and postnatal measures demonstrated adequate psychometric properties. Further studies are needed to determine the most robust perinatal measures for researchers and clinicians.

## Highlights

• Parental perceptions of the parent-infant-bond are important in identifying any difficulties and strengths.
• This is the first comprehensive review to assess the psychometric properties of 14 antenatal and 18 postnatal measures.
• The *Postpartum Bonding Questionnaire* was the most researched measure compared to other measures.
• The administrative properties were good for most measures, suggesting their feasibility, acceptability and attainability.
• Although several studies reported on validity and reliability, most measures lacked adequate methodological quality.

## 1 Introduction

A large body of research now confirms that the early stages of the parent and infant relationship exert an important influence over a child's future development, psychological wellbeing and life chances (Ainsworth, 1979; Bowlby, 1982) and infancy is considered to be the most cost-effective time to intervene (Doyle, Harmon, Heckman, & Tremblay, 2009). Consequently, various organizations and government reports have been advocating the need to address the early stages of parenting with the intent of strengthening the early parent-infant-relationship (e.g., Allen, 2011; Ellyatt, 2017; Moullin, Waldfogel, & Washbrook, 2014; NHS England, NHS Improvement, and National Collaborating Centre for Mental Health, 2018; National Institute for Health and Care Excellence (NICE), 2013, National Institute for Health and Care Excellence (NICE), 2014; Public Health England, 2019; World Health Organization (WHO) and Calouste Gulbenkian Foundation, 2014; Wright et al., 2015). A good early parent-infant-relationship, in which the parents are sensitive and responsive to their infant's physical and emotional needs, lays the foundation for a child's future self-esteem and resilience, their ability to regulate their emotions and their capacity to form close relationships (Bowlby, 1979, Bowlby, 1988; Thompson, 2000; Winston & Chicot, 2016; Wright et al., 2015). Conversely, poor early relationships place children at increased risk of poor cognitive, social and emotional outcomes (Leclére et al., 2014; van Ijzendoorn, Schuengel, & Bakermans-Kranenburg, 1999; Winston & Chicot, 2016; Wright et al., 2015).

Given the importance of the early parent-child-relationship and emotional bond, it is paramount to identify how to support parents in strengthening or improving this relationship effectively when there are any difficulties. An important step in doing so is to be able to identify parents who may be struggling to bond with their developing fetus and/or baby in order to offer them an increased level of support (Royal College of Psychiatrists, 2018). However, the prevalence of difficulties in the early parent-child-relationship can vary depending on how and what is being measured, with some researchers examining bonding via questionnaires (Condon & Corkindale, 1998; van Bussel, Spitz, & Demyttenaere, 2010; Wittkowski, Wieck, & Mann, 2007) and this emotional bond or the reciprocal and interactive relationship between parent and infant often referred to as attachment via observation (Ainsworth, Blehar, Waters, & Wall, 1978; Crittenden, 2001; Bicking Kinsey, & Hupcey, 2013; Lotzin et al., 2015; Noorlander, Bergink, & van den Berg, 2008). Hereby, it is imperative to define the terms ‘bonding’ and ‘attachment’ because they are seen as different concepts but often used synonymously (e.g., Benoit, 2004; Bicking Kinsey & Hupcey, 2013; Redshaw & Martin, 2013). Bonding is described as the tie from the parent to the infant (Bicking Kinsey & Hupcey, 2013; Kennell & McGrath, 2005); it generally consists of feelings and emotions that parents experience towards their infant (Bicking Kinsey & Hupcey, 2013). Attachment is seen as the interplay and reciprocity between the parent and the child (Bicking Kinsey & Hupcey, 2013; Kennell & McGrath, 2005), which usually develops during pregnancy between the parent and the fetus (Condon & Corkindale, 1997). Attachment is part of the parent-child-relationship whereby the parent's role is to ensure the safety, security and protection of the child (Bowlby, 1982). Since the concepts of ‘bonding’ and ‘attachment’ are closely related and have been widely researched, we have opted to include both within the term ‘parent-infant-relationship’.

The ‘gold standard’ for the assessment of parent-child-attachment, and as such the reciprocal aspect of the parent-child-relationship, is via the use of behavioral, observational measures used with parents or other caregivers and their children over 1 year old, such as the Strange Situation task (Ainsworth et al., 1978) and the Attachment Q-Set (Waters & Deane, 1985). Several observational assessment tools exist to evaluate attachment and interaction behaviours between parent and child (up to 30 months old) (e.g., for reviews see Gridley et al., 2019; Lotzin et al., 2015; Mesman & Emmen, 2013; Tryphonopoulos, Letourneau, & Ditommaso, 2014). However, these measures have two key limitations. Firstly, they are time- and resource-intensive and require extensive training to administer and interpret. This limits their use by practitioners, for example, in obstetric, pediatric or primary-care services which mostly lack the time, facilities and training to administer these assessments (van Bussel et al., 2010). Secondly, it has been argued that it is impossible to gain a complete understanding of attachment without also assessing the subjective experience of the parent (Condon, 2012; Scopesi et al., 2004).

Whilst there may always be challenges in enabling parents to disclose any difficulties in bonding or forming emotional ties with their developing fetus or infant to healthcare professionals during routine appointments because parents fear being stigmatised (Morsbach & Prinz, 2006) and/or the involvement of social services and the potential loss of custody (NICE, 2014), developing reliable, valid and sensitive measures may be useful in assisting with the assessment of the early parent-child-relationship and the quest to endorse emotional experiences and beliefs in facilitating parental disclosure.

Furthermore, self-report measures allow us to gain insights into the factors parents perceive to influence their relationship with their child. Given the fact that attachment or bonding in the antenatal period is largely one-sided, consisting mainly of the subjective experiences reported by the parent with little observable behavior (relative to the postnatal period) shown by the fetus, antenatal measures are usually self-reported. Although self-report measures are subject to social desirability bias which can skew interpretation (Arnold & Feldman, 1981; van de Mortel, 2008), they are less costly and labour-intensive to administer (Streiner, Norman, & Cairney, 2015). In addition, they allow us to gain an understanding into the parent's subjective experience of their relationship with their child, which can be meaningful clinically and valuable for research (Condon & Corkindale, 1998; Scopesi et al., 2004). In clinical settings, valid and reliable measures, which are quick and easy to administer, can facilitate screening for difficulties in the parent-infant-relationship and they can also be used to assess change (Brockington et al., 2001). Moreover, the relative ease of administration means that these instruments can be more readily incorporated into large-scale studies and surveys, including those with multiple follow-ups, thereby facilitating research in this area (Pallant, Haines, Hildingsson, Cross, & Rubertsson, 2014). In order to have clinical and research utility, self-report measures must meet criteria for validity and reliability (Crandall, 1976; Streiner et al., 2015) and ideally convergence or concurrent validity with a ‘gold standard’ observational measure. However, when choosing a measure, clinicians or researchers also need to know which measure is suitable for their population and which one accurately assesses change (Streiner et al., 2015), as evidence-based assessment is considered intrinsic to professional practice (e.g., Hunsley & Mash, 2008).

Several parent-report measures of the early parent-infant-relationship have been developed, which differ in terms of their focus, format, content, length, theoretical underpinnings, the purpose for which they were developed, and the extent to which information exists regarding their validity and reliability. Whilst recent reviews have explored the associations between pre-and postnatal bonding (McNamara, Townsend, & Herbert, 2019; Tichelman et al., 2019), only three reviews have explicitly assessed self-report measures of the parent-child-relationship and examined their psychometric properties (Perrelli et al., 2014; Pritchett et al., 2011; Van den Bergh & Simons, 2009). These three reviews differed in their focus. Van den Bergh and Simons (2009) critically evaluated information of the construction and psychometric properties of three maternal-fetal attachment measures only: the *Prenatal Attachment Inventory* (*PAI*, Müller, 1993), the *Maternal-Fetal Attachment Scale* (*MFAS*, Cranley, 1981) and the *Maternal Antenatal Attachment Scale* (*MAAS*, Condon, 1993). Although the PAI and the MFAS appeared to have some robust psychometric properties, all three measures had weaknesses and required further psychometric validation. Pritchett et al. (2011) described the validity and reliability of measures of family functioning. However, the inclusion of 107 measures meant that no measures were reviewed in specific detail. Finally, Perrelli, Zambaldi, Cantilino, and Sougey (2014) undertook an integrative review of measures that could be used in pregnancy and in the first year postpartum. Their review identified 23 articles published after 2002 relating to 13 measures, of which ten were measures completed by parents. Whilst this review identified many of the important and widely used parent-report measures of the early parent-infant-relationship, only a relatively small number of research studies and measures were identified.

A further limitation of these reviews is that they did not use a formal quality appraisal tool which would have allowed for a detailed assessment of the papers' methodological quality and an easier comparison between measures (Terwee et al., 2012). However, a standardized, evidence-based approach to reporting the psychometric properties is essential in order to ensure that the quality of measures used in clinical practice and service improvements is appropriately high (e.g., Kilbourne et al., 2018). Consequently, a review that can be considered a relevant and comprehensive guide for researchers and clinicians is required, especially given the focus on the expansion of perinatal mental health services (NHS England, NHS Improvement, and National Collaborating Centre for Mental Health, 2018).

Taking into consideration the aforementioned gaps in the perinatal field, the main aim of the current systematic review is to provide an overview and evaluation of existing parent- measures of the parent-infant-relationship to assist researchers and clinicians in identifying the most suitable measure to use in their research, practice or service. Specifically, the current review addresses the limitations of previous reviews by bridging the gap between the depth and breadth of the included measures within the systematic review. We aimed to achieve this by a) appraising only measures that assess the parent-infant-relationship in terms of perceived bond or parent-reported attachment rather than broader or related concepts (e.g., maternal self-efficacy, maternal attitudes) in studies that specifically aimed to develop a measure or test its psychometric properties; b) utilising a systematic search strategy to increase confidence that the review included a comprehensive list of measures; c) applying the COnsensus-based Standards for the selection of health Measurement INstruments (COSMIN; Mokkink et al., 2018; Prinsen et al., 2018; Terwee et al., 2018), a comprehensive and systematic tool for appraising and comparing the quality of individual measures in terms of their psychometric properties and clinical utility, and d) identifying relevant administrative properties of the identified measures (Bot et al., 2004).

## 2 Methods

This systematic review was conducted in line with the Preferred Reporting Items for Systematic Reviews and Meta-Analyses (PRISMA) guidelines (Moher et al., 2009) and registered with the PROSPERO database (www.crd.york.ac.uk/prospero; registration number CRD42017078512).

### 2.1 Search strategy and paper inclusion

In keeping with the aims of the review, we conducted a systematic literature search in eight electronic databases using four steps. In step 1 we designed a search strategy to retrieve peer-reviewed papers relevant to the development, validation and implementation of self-report measures of the early parent-infant-relationship, which was piloted by one reviewer (CG). The aim of this pilot search was to increase specificity and sensitivity to capture the highest possible proportion of relevant articles. This pilot search resulted in low specificity with too many irrelevant articles being initially retrieved; thus, following further consultation with a university librarian, we refined our search strategy by adding the ‘adjacency’ operator (abbreviated as ‘ADJn’ whereby *n* refers to a number of words from each other in any order) to our search terms. This strategy led to step 2 whereby another reviewer (SV) conducted the search in three electronic platforms (Ovid, Clarivate and EBSCO) and their eight bibliographic databases from their inception to the end of April 2019: MEDLINE, Embase, PsycINFO, PsycARTICLES, Maternity and Infant Care, Health and Psychosocial Instruments, Web of Science and CINAHL. The search was updated in August 2019.

Limitations for language or year of publication were not set because the exclusion of non-English studies could introduce risks of bias and, therefore, each non-English study should be evaluated case-by-case to maintain internal validity (Higgins & Green, 2011; Neimann Rasmussen & Montgomery, 2018).

In step 2, we searched the following terms in the title, abstract or keywords in those eight databases (see Appendix A for a sample search strategy): 1) (parent*or maternal or paternal or mother* or father*) adj7 (child or infant or newborn or foet* or fetus or fetal or baby or neonate); 2) (antenat* or prenat* or puerper* or postnat* or postpart* or peripartum or pregnan* or perinat*); 3) (measur* or scale$ or questionnaire$ or construct$ or tool$ or inventor* or instrument$ or test*) adj7 (attachment or relation* or bond* or orientation or synchrony or synchronicity or “emotional availability” or attitude* or belief* or responsiv* or feel* or interact*). Papers retrieved from this search were then screened for measures relevant to the aims of the review.

In step 3, further searches with the names of identified measures were conducted in a ninth database (i.e., PubMed) to identify the original development/validation paper(s) for that measure as well as papers reporting further validation work undertaken with any identified and included measures. In the final and fourth step, the reference lists of included articles were checked for additional relevant studies. When the initial development/validation work for a measure was unpublished, further information was sought from study authors. When this was not possible, we extracted relevant development and validation process information about this measure from papers by the original authors.

To verify inter-rater reliability of the screening, an independent research assistant (CS) independently double-screened 1% of all identified articles during the screening stage and 20% of potentially eligible articles to determine their inclusion or exclusion. The percentage of inter-rater agreement and Cohen's kappa were calculated on both types of screening to ensure the validity of the screening process.

### 2.2 Inclusion criteria of papers and article selection

Papers were included if they described the initial development and validation of a relevant measure. Papers were also included if they described an attempt to validate and/or to test the psychometric properties of an included measure, and this was the clearly stated aim of the paper. Decisions about the inclusion/exclusion of measures and papers were based on the initial judgment of two reviewers (AW and CG). Their decisions were verified by two other reviewers (SV and AM), and any disagreements were resolved through consultation with the fifth reviewer (MH).

### 2.3 Inclusion and exclusion criteria of measures

Measures were included if they were completed by the parent and assessed the parent's perception of the parent-infant-relationship or bond during the antenatal period or the postnatal period up until an infant age of two years. Measures were excluded if they were not assessing the parent-infant-relationship per se but instead assessed a related concept (e.g., ‘parenting style’ or ‘attitudes to pregnancy’) or if they only assessed the parent-infant-relationship as part of a subscale in a longer inventory (e.g., the *MAMA,* Kumar, Robson, & Smith, 1984). As the content of measures assessing related constructs (e.g., maternal self-efficacy, maternal attitudes, etc.) can be very similar to those of measures explicitly described by authors as measures of bonding or attachment, we based inclusion decisions on item content rather than author description (e.g., the *How I Feel About My Baby Now Scale, FAB*, Leifer, 1997; the *Mothers' Object Relations Scales Short Form, MORS-SF,* Oates, Gervai, Danis, Lakatos, & Davies, 2018).

On several occasions, original measure authors or other researchers proposed shortened or alternative versions of measures which had already been identified and included in the current review. For example, we included the *Short Postpartum Bonding Questionnaire* (*SPBQ*; Kinsey, Baptiste-Roberts, Zhu, & Kjerulff, 2014), which was based on the original 25-item *Postpartum Bonding Questionnaire* (*PBQ*; Brockington et al., 2001) but shortened to address the need for a briefer instrument to measure parent-infant-bonding as part of a large scale telephone interview survey. Similarly, in several cases, researchers (but not the original authors) conducted psychometric testing on slightly different versions of measures (e.g., containing fewer items or having fewer Likert response categories). These alternative versions were also included in the current review and treated as separate independent measures.

### 2.4 Assessing the psychometric properties of included measures

We evaluated the measurement properties of the included studies and measures using: 1) the COSMIN criteria for evaluating the quality of the measure development studies and content validity studies (Terwee et al., 2018), 2) the COSMIN Risk of Bias checklist (Mokkink et al., 2018) to assess the methodological quality of the studies, 3) the COSMIN checklist to examine eight psychometric results, including structural validity, internal consistency, reliability, hypothesis testing for construct validity, cross-cultural validity/measurement invariance, measurement error, criterion validity and responsiveness (Mokkink et al., 2018; Prinsen et al., 2018), and 4) the modified Grading of Recommendations Assessment, Development, and Evaluation (GRADE) approach to examine the quality of the evidence (Mokkink et al., 2018). All materials are available at www.cosmin.nl/index.html.

#### 2.4.1 Step 1: Quality assessment of included studies

The first step in the process to assess the methodological quality of included studies is achieved via the application of the ‘COSMIN Risk of Bias checklist’ (Mokkink et al., 2018). This checklist consists of categories for appraising the quality of the outcome measure development studies as well as the quality of various psychometric measurement properties which are outlined above (see Table 1 for definitions of measurement properties, Mokkink et al., 2018). Content validity was assessed in terms of relevance, comprehensiveness and comprehensibility of the measure's items (Terwee et al., 2018).

**Table 1:**
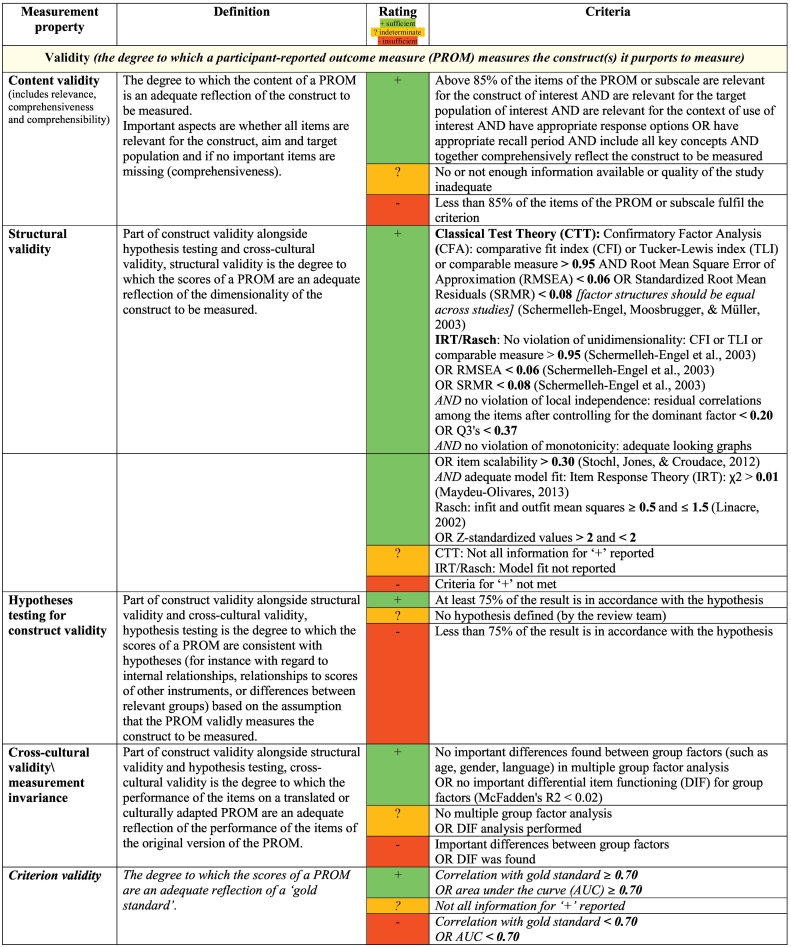

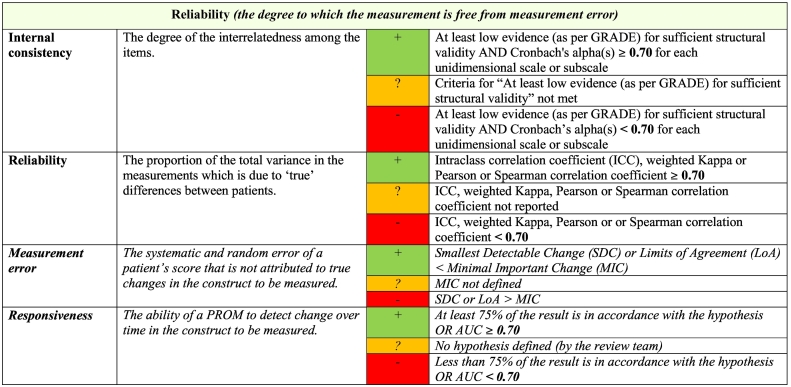
Definitions and criteria for good measurement properties^⁎^.

During the pilot stage we identified that many researchers did not explicitly describe what they explored or evaluated in terms of content validity in their studies. Consequently, we expanded the COSMIN's definitions of ‘relevance’ and ‘comprehensibility’: studies were considered to evidence ‘relevance’ when they evaluated the relevance, appropriateness, suitability and/or acceptability of each item in the target population. In terms of ‘comprehensibility’, studies were rated if they evaluated the understanding, coherence, clarity, meaning and/or ambiguity of the items and whether the response options, instructions and/or the recall period were clear and comprehensible. ‘Comprehensiveness’ was evaluated in accordance with the COSMIN guidelines whereby participants should have been explicitly asked about whether the items comprehensively covered the construct that the outcome measure (or the sub-scale) intended to measure or if the included domains together comprehensively covered the wider construct measured by the total score of the outcome measure (Terwee et al., 2018).

Each measurement property (including content validity) was rated across several items assessing different aspects of quality, using a four-point COSMIN Risk of Bias scale (i.e., 4 = ‘very good’, 3 = ‘adequate’, 2 = ‘doubtful’, 1 = ‘inadequate’). An overall score for the methodological quality of a study was determined for each measurement property separately by taking the lowest rating of any of the items in a given category. When the developers of the original version of a measure omitted to provide detailed information on one or more psychometric properties, but there was sufficient information to assume that the study was conducted adequately, we deviated from the stricter COSMIN guidance and opted to give an ‘adequate’ or ‘doubtful’ rating rather than an ‘inadequate’ rating. For example, if in the measure development study it was unknown whether the qualitative data, collected for the purposes of cognitive interview or pilot testing, were coded by one or two researchers independently, we rated it as ‘doubtful’ rather than ‘inadequate’ due to lack of information.

Interpretability or the degree to which one can assign qualitative meaning to quantitative scores (Mokkink et al., 2010, Mokkink et al., 2009) is not considered a measurement property but an important characteristic of a measurement instrument (Mokkink et al., 2018). This means that investigators should provide information about clinically meaningful differences in scores between subgroups, floor and ceiling effects, and the minimal (clinically) important change (Mokkink et al., 2009). However, since a limited number of studies reported aspects of interpretability, we could not present this in our review.

#### 2.4.2 Step 2: assessment of study outcomes

The second step involved assessing the study results for each of the included measures, according to the updated 2018 measurement for good measurement properties (Mokkink et al., 2018; Prinsen et al., 2018). These criteria cover eight measurement properties, for each of which the rater is required to assign ‘+’, ‘?’ or ‘–’. A ‘+’ is assigned when the study findings provide good evidence of a measure exhibiting this property (i.e., ‘sufficient’ rating); a ‘?’ is assigned when results are equivocal or appropriate tests have not been performed (i.e., ‘indeterminate’ rating) and a ‘–’ is assigned when appropriate tests have been performed and the result suggests that the measure does not exhibit this property as defined by the checklist criterion (i.e., ‘insufficient’ rating). This checklist and quality criteria are presented in Table 1.

The content validity of an outcome measure was evaluated according to the quality and results of the available studies and the outcome measure itself (Terwee et al., 2018). Although the COSMIN guidelines suggest not to rate a study if the quality of the study (according to the risk of bias assessment) was ‘inadequate’, we decided to rate all studies, including those with an ‘inadequate’ quality rating, in order to gain a comprehensive overview of a particular outcome measure. Content validity of each outcome measure was rated according to the development studies (scored as ‘+’, ‘?’ or ‘–’ for ‘sufficient’, ‘indeterminate’ and ‘insufficient’ ratings, respectively), available content validity studies (also scored as ‘+’, ‘?’ or ‘–’) and ratings given by two reviewers (AW and SV) (scored as ‘+’, ‘±’ or ‘–’ for ‘sufficient’, ‘inconsistent’ and ‘insufficient’ ratings, respectively). When no content validity studies were available or only content validity studies of inadequate quality were available, the overall ratings for content validity were determined according to the reviewers' ratings as per COSMIN criteria.

In order to rate the structural validity of measures, we had to adapt the criteria as the current 2018 COSMIN criteria for good measurement properties do not include guidance for rating the results of Exploratory Factor Analysis (EFA). Consequently, EFAs were rated as ‘sufficient’ if ≥50% of the variance was explained (as in previous versions of the COSMIN criteria; see Terwee et al., 2012). Such evidence was downgraded for methodological quality based on the risk of bias checklist (i.e., studies using EFA can only be rated as ‘adequate’ rather than ‘very good’). When the % of variance accounted for (in the case of EFA) or model fit statistics (in the case of CFA) were not reported, an ‘indeterminate’ rating was assigned. Finally, when higher quality evidence (e.g., CFA) was available for a given measure, lower quality evidence (e.g., EFA) was ignored.

In terms of hypothesis testing for construct validity, the decision was made to include any published measure as a comparator instruments that measured a similar construct (e.g., other attachment measures included in the current review or a subscale from a measure not included in the review, such as the attitudes towards pregnancy and the baby subscale of the *MAMA* scale, Kumar et al., 1984). To receive a ‘sufficient’ rating, 75% of the correlations tested had to meet the cut-off of *r* ≥ 0.50 against a comparator instrument measuring a similar construct. Given the lack of an established self-report measure for the construct under study, caution is needed in interpreting the results for this measurement property.

We also adapted the criteria for rating reliability due to ambiguity in the COSMIN guidelines in a way that the studies that reported a correlation coefficient (i.e., Pearson's or Spearman's) but did not report the intraclass correlation coefficient (ICC) for (test-retest) reliability would still receive a ‘sufficient’ or ‘insufficient’ rating which would normally receive an ‘indeterminate’ rating if the ICC was not applied. Instead, we decided to reflect this in the quality of evidence (described in Section 2.4.3) whereby studies that did not use the more robust method (i.e., the ICC) would get a lower rating even if the study received a ‘sufficient’ rating on reliability.

#### 2.4.3 Step 3: summary and quality grading of the evidence

As per COSMIN criteria, the psychometric findings reported in each of the included studies were summarized and graded for each measure. This process resulted in each measure being assigned two ratings: 1) an overall rating of ‘sufficient’ (‘+’), ‘insufficient’ (‘–’) or ‘indeterminate’ (‘?’) for the eight psychometric properties (except content validity), or an overall rating of ‘sufficient’ (‘+’), ‘insufficient’ (‘–’) or ‘inconsistent’ (‘±’) for content validity, and 2) an overall rating of methodological quality for each measurement property (‘high’, ‘moderate’, ‘low’ or ‘very low’). The latter rating is achieved through following the modified GRADE approach, which involves consideration of several factors in rating methodological quality of the pooled results (e.g., the evidence for risk of bias, inconsistency of results and imprecision through small sample sizes). Importantly, this approach also takes into account the number of available studies and the methodological quality of each individual study. More detailed information on how GRADE was conducted can be found in the COSMIN manual (Mokkink et al., 2018; Prinsen et al., 2018; Terwee et al., 2018). Definitions for each of the GRADE quality level ratings are shown in Table 2.

**Table 2:**
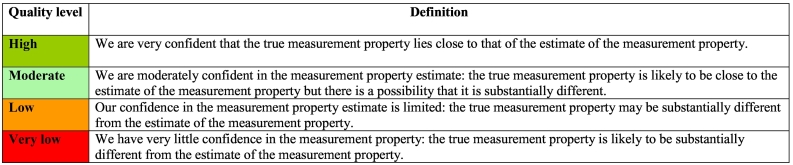
Definitions of quality levels using the GRADE approach.

Notes: Estimate of the measurement property refers to the pooled or summarized result of the measurement property of a given measure. These definitions were adapted from the GRADE approach.

#### 2.4.4 Step 4: assessment of practical administrative properties

Practical, administrative or clinimetric properties that would affect the ease with which each of the measures could be employed in a clinical or research context, but are not covered in the COSMIN or Terwee et al. (2018) checklists, were also assessed*.* The following properties were assessed:

1. *Time to administer:* As measure authors did not routinely report this property, this was assessed independently by two reviewers (SV and AM) who completed each measure and timed themselves. As per Bot et al.'s (2004) clinimetric checklist, a positive rating was given when the questionnaires could be completed within 10 min.
2. *Ease of scoring* refers to the extent to which the measure can be scored by a trained investigator or expert. In accordance with Bot et al.'s (2004) checklist, the scoring method was rated as easy when the items were simply summed, moderate when a visual analogue scale or simple formula was used and difficult when either a visual analogue scale in combination with a formula or a complex formula was used.
3. *Readability and comprehension:* The Flesch Reading Ease (FRE; Flesch, 1948) method was used to assess readability and comprehension. The text is rated on a 100-point-scale in which 100 represents the easiest text and 0 the hardest. Measures scoring ≥90 using the FRE were considered excellent for this property; measures scoring between 80 and 89 were considered good; measures scoring between 70 and 79 were considered fair and measures with scores below <69 were considered poor.
4. *Availability and conditions of use* refers to the ease with which researchers/clinicians can obtain the questionnaire and whether it is free to use. If the measure was easily accessible on the internet or through e-mailing the first author, and it was also free to use, availability was classed as excellent. If a measure was difficult to obtain but was free of cost, the measure was classified as good. If a measure was easy to obtain but had a cost, the measure was classed as fair. Finally, if a measure was difficult to obtain and there was a cost for accessing or utilising the instrument, the measure was classified as ‘poor’.

### 2.5 Inter-rater reliability

Extraction of data and the assessment of the methodological quality (i.e., risk of bias) was performed by reviewers independently (AM, SV and CG). The assessment of all psychometric properties (except content validity) was performed by one reviewer (AM). The reliability of ratings was confirmed by having another reviewer (SV), who independently rated 20% of the papers. For the measure development studies and content validity studies, another reviewer (SV) completed risk of bias and rated content validity; 20% of those papers were independently rated by an independent research assistant (CS). Inter-rater reliability was met if Cohen's kappa between the two reviewers was above 0.61, indicating ‘substantial’ agreement (McHugh, 2012), on all psychometric ratings. When this was not achieved, the disagreements were discussed and resolved through consultation with another reviewer (AW).

## 3 Results

### 3.1 Review process

The original search identified 15,924 papers. After removing duplicates, the titles and abstracts of 12,081 papers were screened. The titles, abstracts and/or full texts of 220 papers were examined against inclusion and exclusion criteria. In August 2019 the search was repeated which resulted in 600 hits between January and August 2019 which were fully screened. Only two studies were identified: a study describing the development of the MAAS-13 and PAAS-13 (Göbel et al., 2019) which was included in the review and a non-English study describing a Slovenian version of the *PAI* (presented in Appendix B alongside other non-English papers). The agreement for the screening of 1% of all identified articles was 94.7% (kappa = 0.90) and for the 20% of potentially eligible articles the agreement was 75% (kappa = 0.58). Any discrepancies in the exclusion and inclusion of studies were resolved among all reviewers through discussion.

**Fig. 1:**
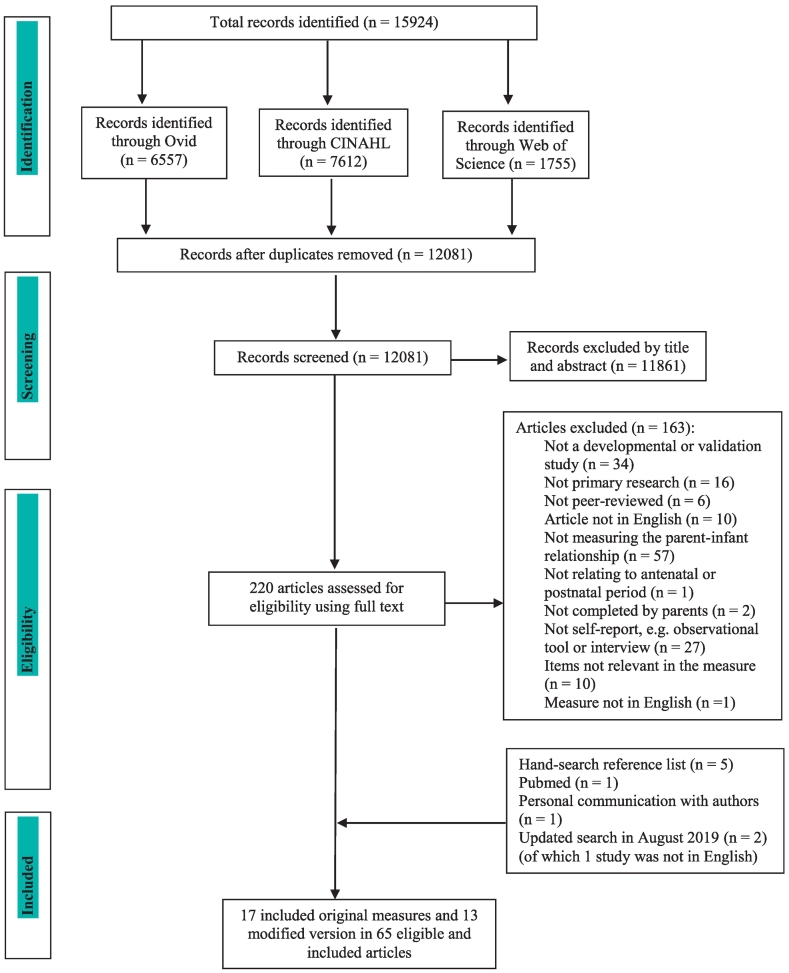
Flowchart of paper selection based on PRISMA guidance.

After a detailed assessment, 65 papers evaluating 17 original measures in associated development studies and 13 modified versions, derived from only four of the identified 17 measures, were included in the review (for the references of the included papers, please see Appendix C). In total, 14 antenatal measures (eight original and six modified versions) and 18 postnatal measures (ten original measures plus eight modified version), of which one measure (the *Prenatal and Postnatal Bonding Scale, PPBS*, Cuijlits et al., 2016*)* could be used antenatally and postnatally, were reviewed. The majority of these measures were maternal, but we also identified four paternal measures (three antenatal and one postnatal version). The search process and outcome are illustrated in Fig. 1.

### 3.2 Study characteristics and information on measure development

The publication dates of the studies describing the original 17 measures ranged from 1977 to 2018. The validation work undertaken for the original 17 measures included studies conducted in eight different countries, such as the USA (*n* = 5), Australia (*n* = 4), the UK (n = 4), the Netherlands (*n* = 1), Hungary (*n* = 1), Korea (*n* = 1), Sweden (*n* = 1) and India (*n* = 1); however, the sample in the *MORS-SF* (Oates et al., 2018) scale development comprised British and Hungarian mothers (see Table 3 for details). Study sample sizes reported by measure authors in their development studies ranged from 19 to 1050 women and 100 to 461 men. The majority of studies included non-clinical samples (*n* = 15), with only two studies using a clinical sample of women with mental illness (Brockington et al., 2001; Hackney, Braithwaite, & Radcliff, 1996).

**Table 3:**
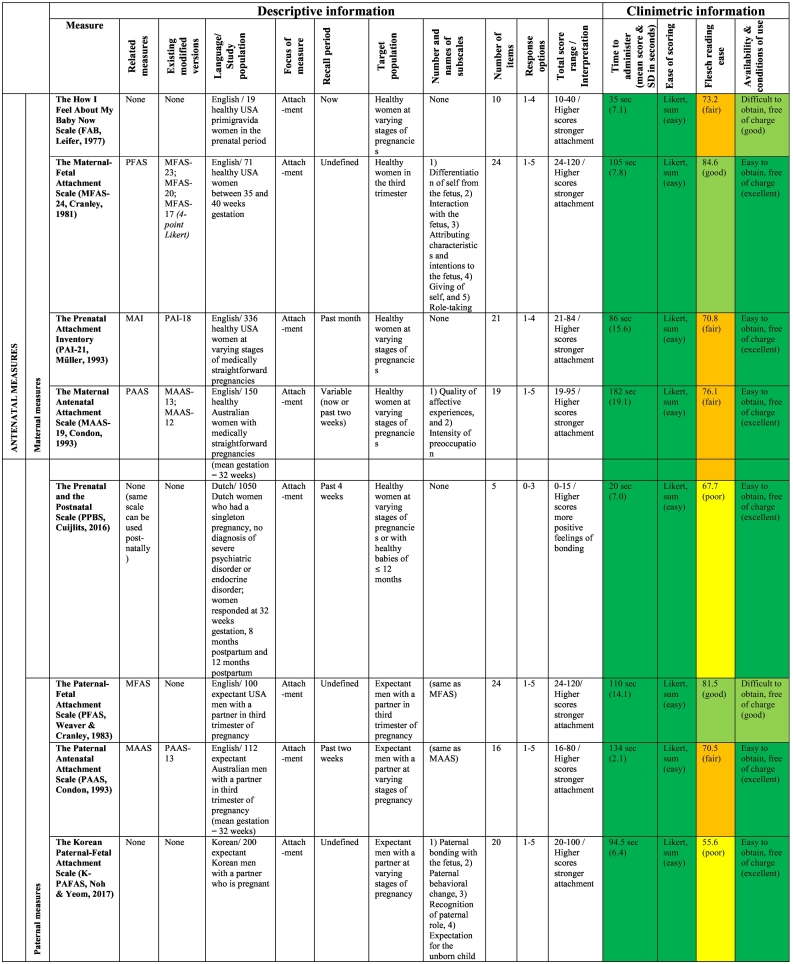

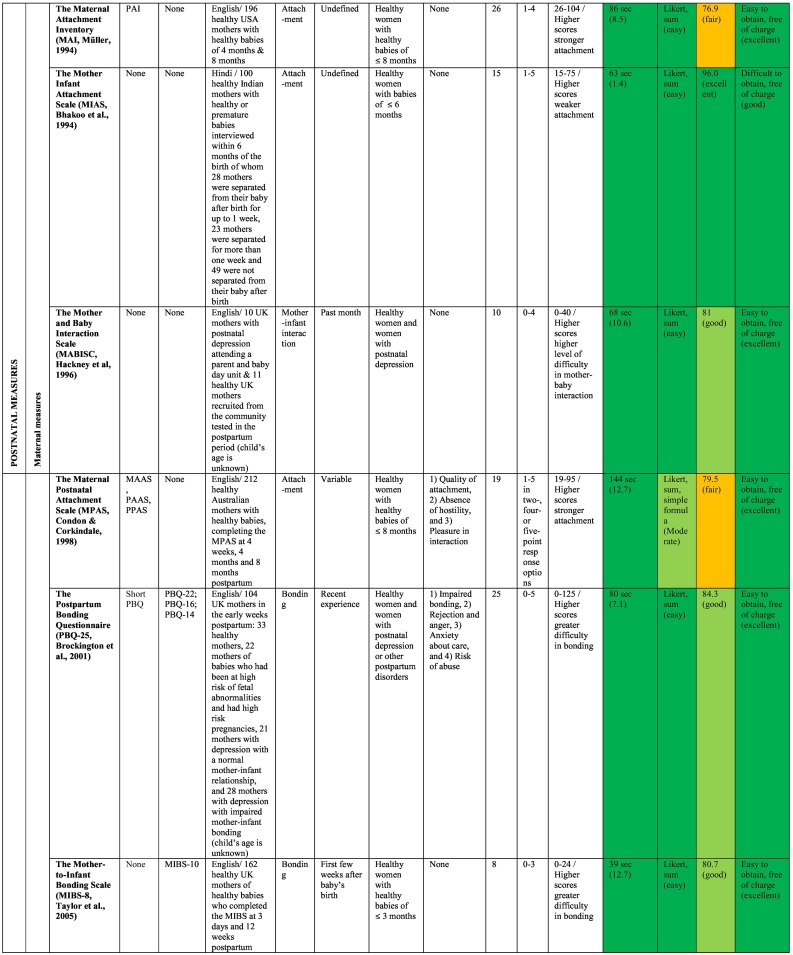

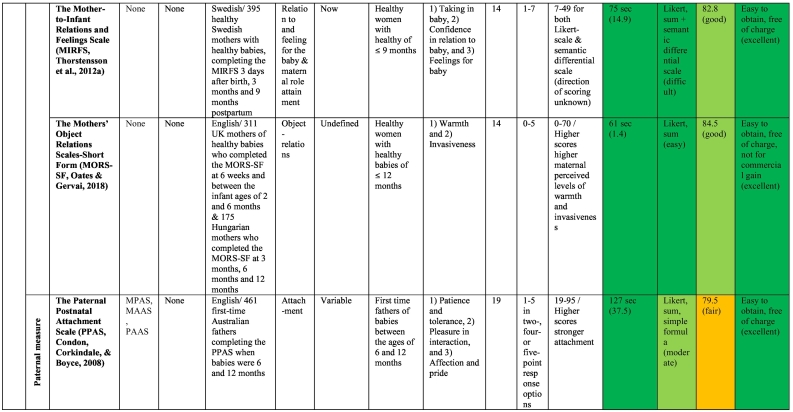
Overview of the included measures and summary of their administrative and clinimetric properties.

### 3.3 Description of identified measures

A description of each of the 17 original measures is presented in Table 3. The majority of measures focused on the assessment of parent reported perceptions of bonding and attachment. However, one measure also focussed on the psychodynamic concept of object relations (i.e., the *MORS-SF,* Oates et al., 2018) and another measure also focussed on the assessment of maternal role attainment (i.e., the *Mother-to-Infant Relations and Feelings Scale, MIRFS*, Thorstensson et al., 2012). Although the authors did not set out to test the *MIRFS*, we included it in this review because Ekström and Nissen (2006) described the *MIRFS*' development and evaluated its content validity.

Eight scales were measures of the parent-fetus-relationship, administered to expectant women and men in the antenatal period of pregnancy: the *How I Feel About My Baby Now Scale* (*FAB*, Leifer, 1997), the *Maternal-Fetal Attachment Scale* (*MFAS*, Cranley, 1981), the *Prenatal Attachment Inventory* (*PAI*, Müller, 1993), the *Maternal Antenatal Attachment Scale* (*MAAS*, Condon, 1993), the *Pre- and Postnatal Bonding Scale (PPBS*, Cuijlits et al., 2016). the *Paternal-Fetal Attachment Scale* (*PFAS*; Weaver & Cranley, 1983), the *Paternal Antenatal Attachment Scale* (*PAAS*; Condon, 1993) and the *Korean Paternal-Fetal Attachment Scale* (*K-PAFAS;* Noh & Yeom, 2017.

As per COSMIN criteria, any modified versions of measures were reviewed separately even if they differed from the original scale by only one item. Three modified versions were identified for the original 24-item-*MFAS* offering different item totals: the *MFAS-23* (Müller, 1993; Müller & Ferketich, 1993), the *MFAS-20* (Busonera, Cataudella, Lampis, Tommasi, & Zavattini, 2016), and the *MFAS-17* (Seimyr, Sjögren, Welles-Nystrom, & Nissen, 2009; Sjögren, Edman, Widstrom, Mathieson, & Uvnas-Moberg, 2004). The original 19-item-*MAAS* had also been shortened in modified versions referred to as the *MAAS-13* (Göbel et al., 2019) and *MAAS-12* (Navarro-Aresti, Iraurgi, Iriarte, & Martinez-Pampliega, 2016).

Five original and five modified versions of these were measures of the mother-fetus-relationship: the *FAB*, the *MFAS-24*, the *MFAS-23*, the *MFAS-20*, the *MFAS-17*, the *PAI-21*, the *MAAS-19, the MAAS-12,* the *MAAS-12* and the *PPBS*. Only three measures assessed the father-fetus-relationship, namely the *PFAS,* the *PAAS* and the *K-PAFAS.* The *PAAS* has also been revised and shortened to the 13-item-*PAAS* (Göbel et al., 2019).

Ten original scales and eight modified versions were measures of the parent-infant-relationship. As can be seen in Table 3, the original scales were the *Maternal Attachment Inventory* (*MAI-26*; Müller, 1994), the *Mother Infant Attachment Scale* (*MIAS*; Bhakoo, Pershad, Mahajan, & Gambhir, 1994), the *Mother and Baby Interaction Scale* (*MABISC*; Hackney et al., 1996), the *Maternal Postnatal Attachment Scale* (*MPAS*; Condon & Corkindale, 1998), the *Postpartum Bonding Questionnaire* (*PBQ-25*; Brockington et al., 2001), the *Mother-to-Infant Bonding Scale* (*MIBS-8;* Taylor, Atkins, Kumar, Adams, & Glover, 2005), the *Mother-to-Infant Relations and Feelings Scale* (*MIRFS*; Thorstensson et al., 2012), the *PPBS* (Cuijlits et al., 2016), the *Mothers' Object Relations Scales Short Form* (*MORS-SF*; Oates et al., 2018) and the *Paternal Postnatal Attachment Scale* (*PPAS*; Condon, Corkindale, & Boyce, 2008). Only the *PPAS* was designed to assess the father-infant-relationship. Of these measures, only the *PPBS* could be used both antenatally and postnatally.

The *MFAS* (Cranley, 1981) and the *PFAS* (Weaver & Cranley, 1983) as well as the *MAAS* (Condon, 1993) and the *PAAS* (Condon, 1993) are maternal and paternal versions of the same measures, respectively, and can be completed by mothers and fathers in the same family. The *MAI* (Müller, 1994) is the postnatal version of the *PAI* (Müller, 1993). Condon and colleagues (Condon, 1993; Condon et al., 2008; Condon and Corkindale, 1997, Condon and Corkindale, 1998; Condon, Corkindale, Boyce, & Gamble, 2013) have produced measures based on Condon's (1993) model of human attachment with antenatal and postnatal measures for both mothers and fathers (i.e., *MAAS, PAAS, MPAS* and *PPAS*).

Of the postnatal measures, the *PBQ* has received the most attention by other researchers who have produced shorter versions, including the *PBQ-22* (Wittkowski, Williams, & Wieck, 2010), the *PBQ-19* (Vengadavaradan, Bharadwaj, Sathynarayanan, Durairaj, & Rajaa, 2019), the *PBQ-16* (Reck et al., 2006), the *PBQ-16-J* (Kaneko & Honjo, 2014), the *PBQ-14* (Suetsugu, Honjo, Ikeda, & Kamibeppu, 2015) and the *Short PBQ* with 10 items (Kinsey et al., 2014). Although the *PBQ* has also been used with fathers at 2 months postpartum in a Swedish study (Edhborg, Matthiesen, Lundh, & Widström, 2005), the *PBQ* was not specifically developed to be used with fathers. As Edhborg et al. (2005) did not evaluate the psychometric properties of the *PBQ* in their male sample, this study was not rated in our review.

The only other postnatal measure with modified versions is the 8-item-*MIBS*, which had been reduced to seven items in the *MIBS-J-7* (Ohara et al., 2016) and extended to 10 items in the *MIBS-J-10* (Yoshida, Conroy, Marks, & Kumar, 2012).

#### 3.3.1 Additional information on the measures' items and target population

The majority of the measures comprise items that are worded as statements on a Likert-scale that typically enquire how the mother is feeling towards the developing fetus or the newborn. For example, the *PAI* includes items, such as “I stroke the baby through my tummy” or “I enjoy feeling the baby move”. The *PBQ* includes items, such as “I feel close to my baby” or “My baby winds me up”, and the *MPAS* includes items, such as “When I am not with the baby, I find myself thinking about the baby: …” or “Taking care of this baby is a heavy burden of responsibility. I believe this is: …”. Only one measure, the *MIRFS*, was a two-part measure in which seven items (worded as statements) evaluated the mothers' perception about the relationship between the mother and her baby (e.g., “I talk a lot with my baby” and “I do not talk at all with my baby”) and seven items (worded as adjectives) explored the mothers' current feelings towards the baby (e.g., “Difficult” and “Easy”). Although most studies described the population with whom the study was conducted, the majority of the studies did not specify the target population of parents by providing information about the gestation age or the infant's age. Consequently, it was impossible to determine the measures' applicability to parents of infants at different developmental ages and we had to assume that they targeted parents of children younger than two years old.

The number of items used in the original 17 measures ranged from five (e.g., the *PPBS)* to 26 items (e.g., the *MAI*). Of the original 17 measures, seven measures were unidimensional (*FAB, PAI, MAI, PPBS, MABISC, MIBS-8* and *MIAS)*, whereas ten measures included multiple sub-scales (*MFAS, PFAS, MAAS, PAAS, K-PAFAS, MPAS, PPAS, PBQ, MORS-SF* and *MIRFS*), which ranged from two (e.g., *MAAS, PAAS* and *MORS-SF*) to five subscales (e.g., *MFAS*).

Most measures were designed for the assessment of parents within a non-clinical population who were asked to reflect on their feelings or thoughts in the present moment (e.g., the *FAB*), the past two weeks (e.g., the *PAAS*) or during the past month (e.g., the *PPBS*, the *PAI-21,* the *MABISC*). However, six measures did not state a specific recall time and three measures accepted a variable timeframe.

As our search did not exclude studies not written in English initially, our review identified that most of the 17 original and 13 modified measures are available in a total of 17 languages including English. Other language versions included measures in Chinese, Korean, Japanese, German, Italian, French, Portuguese, Spanish, Dutch, Swedish, Norwegian, Hungarian, Turkish, Persian, Tamil and Hindi. Four measures were only available in one language: the *FAB* in English only, the *MIAS* in Hindi only, the *K-PAFAS* in Korean only and there appears to be only a Swedish version of the *MIRFS*. In addition, the *MORS-SF* was validated on a mixed sample of British and Hungarian women.

Although it was our original intention to include measures not written in English, we were unable to a) apply the COSMIN criteria consistently across these studies ourselves and b) identify professional translators trained in the application of the COSMIN criteria for the foreign language papers we identified. For comprehensiveness, the foreign language papers are presented in Appendix B.

### 3.4 Measurement properties assessed

Sixty-five studies pertaining to the 17 measures and their 13 modified versions rated aspects of validity and reliability (see Table 4 and Table 5). Several of the included studies (e.g., Bienfait et al., 2011; Brockington et al., 2006, Brockington et al., 2001) tested for diagnostic accuracy (i.e., sensitivity and specificity of the measure in detecting bonding difficulties), but this property does not fall within the COSMIN taxonomy and consequently it was not rated.

**Table 4:**
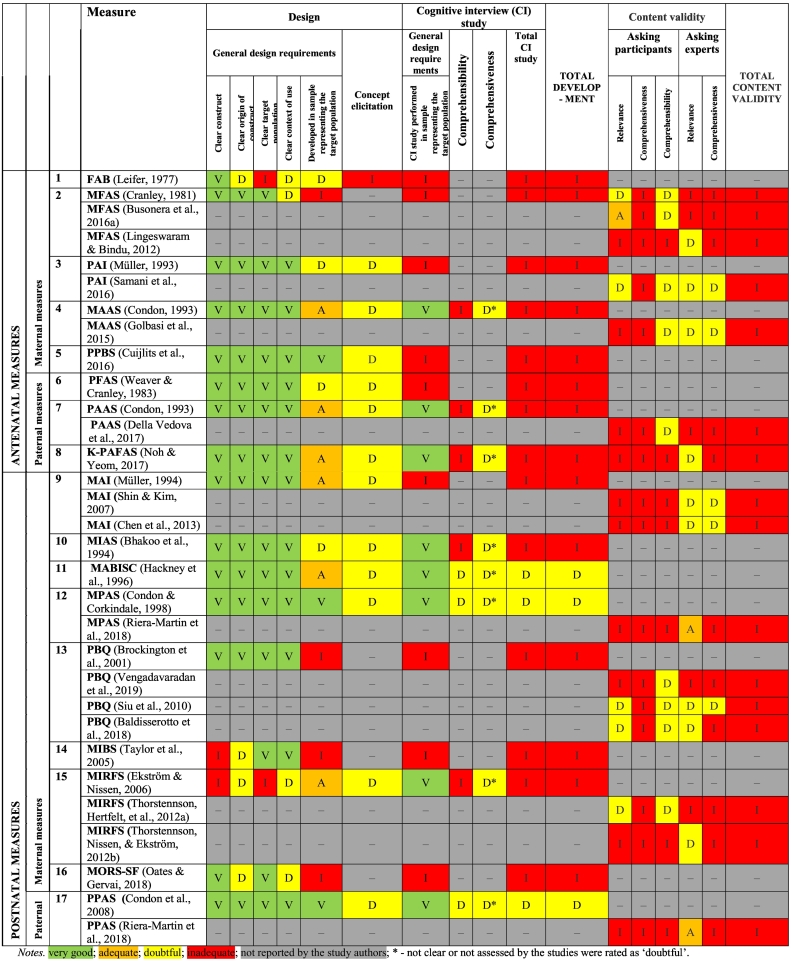
Quality of the measure development (*n* = 17 measures) and content validity (*n* = 16 measures) (Baldisserotto et al., 2018; Chen et al., 2013; Della Vedova and Burrp, 2017; Golbasi et al., 2015; Linacre, 2002; Lingeswaran and Bindu, 2012; Maydeu-Olivares, 2013; Riera-Martin et al., 2018; Schermelleh-Engel et al., 2003; Shin and Kim, 2007; Siu et al., 2010; Stochl et al., 2012; Yoshida et al., 2012).

**Table 5:**
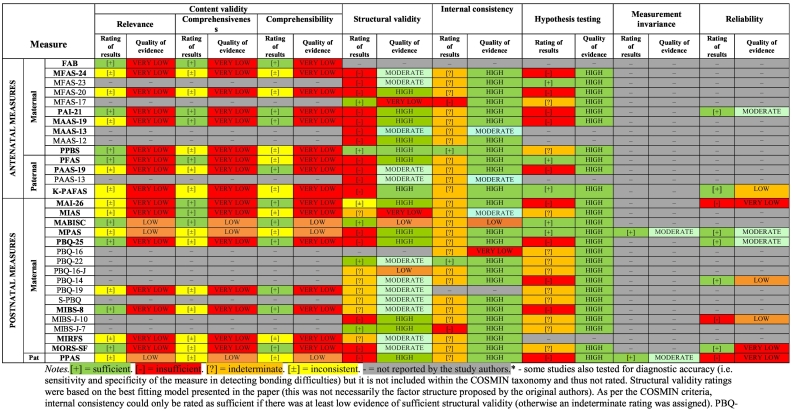
Synthesis of psychometric properties and quality of evidence (using GRADE)^⁎^.

#### 3.4.1 Assessment of validity

##### 3.4.1.1 Content validity

According to the COSMIN criteria for assessing content validity (Terwee et al., 2018), the relevance, comprehensiveness and comprehensibility of the 17 measures and their 13 modified versions were rated separately in a multi-step process. Firstly, the overall quality of the development (i.e., risk of bias checklist) of the 17 original outcome measures were evaluated: three original measure development studies (*MABISC, MPAS* and *PPAS*) had a ‘doubtful’ rating and 14 original measure development studies were rated as ‘inadequate’ (see detailed ratings in Table 4). In this step, the quality of the content validity studies was also evaluated according to whether participants in a content validity study had been asked about the relevance, comprehensiveness and comprehensibility of the measure items and whether professionals had been asked about the relevance and comprehensiveness of the measure items.

A total of 16 out of 65 studies (Table 4) evaluated content validity either among a participant group (i.e., mothers or fathers) or among a professional group (i.e., midwives, psychologists, psychiatrists, researchers, etc.). ‘Relevance’ and ‘comprehensibility’ was evaluated among participants in six and nine studies, respectively, and among professionals in 10 and five studies, respectively. However, none of the content validity studies evaluated ‘comprehensiveness’ among participants. From all content validity studies evaluating relevance, comprehensiveness and comprehensibility among participants and/or professionals, only two studies received an ‘adequate’ rating: Busonera et al. (2016a) for ‘relevance’ among participants using the *MFAS* and Riera-Martin et al. (2018) for ‘relevance’ among professionals on the *MPAS* and the *PPAS*. The remaining studies received a ‘doubtful’ rating for ‘relevance’, ‘comprehensibility’ and/or ‘comprehensiveness’ when these properties were assessed in a particular study (see Table 4 for detailed ratings).

Once the quality of the development studies and content validity studies were rated for each measure, the content validity of an outcome measure was evaluated. With regards to the 17 original measures, the overall content validity was rated as ‘sufficient’ (‘+’) for the following measures: the *FAB,* the *PAI* and the *MABISC.* However, the remaining 14 measures were rated to have ‘inconsistent’ (‘±’) evidence for their overall psychometric properties (see Appendix D for detailed ratings). The reasons for ‘inconsistent’ ratings were explored and in the majority of the cases, the information on relevance, comprehensiveness and comprehensibility presented by the measure authors in the papers was poor. However, the independent evaluation by two reviewers of the scale alone indicated that the information given was ‘sufficient’ and met the COSMIN criterion of ‘+’.

In the third step, the quality of the evidence was rated using the GRADE approach. As the development study of the measure received ‘inadequate’ quality ratings, according to COSMIN criteria these studies have to receive a lowered rating in terms of the GRADE. Therefore, three scales (*MABISC, MPAS* and *PPAS*) were rated as ‘low’ and 14 measures were rated as ‘very low’ for relevance, comprehensiveness and comprehensibility according to the GRADE approach (Table 5).

##### 3.4.1.2 Structural validity (part of construct validity)

In order to rate structural validity, we adapted the 2018 COSMIN criteria for good measurement properties as previously outlined, because many of the included studies conducted Exploratory Factor Analysis (EFA); however, the COSMIN criteria does not provide guidance for rating the results of the EFA.

Structural validity was tested for the majority of the included measures (27 out of 29; 93%), but not for the *FAB* or the *PBQ-16*. Of the prenatal measures, only the *MFAS-17* and the *PPBS* were assigned a ‘sufficient’ rating. The remaining prenatal measures were assigned ‘insufficient’ ratings. Of the postnatal measures, the *MABISC,* the *PBQ-22,* and the *MIBS-J-7* were assigned ‘sufficient’ ratings, whereas the *MPAS,* the *PBQ-25,* the *MIBS-J-10,* the *MORS-SF* and the *PPAS* were assigned ‘insufficient’ ratings. The *MAI-26* was assigned an ‘inconsistent’ rating because there was evidence for structural validity but for its two different factor structures. The remaining postnatal measures were assigned ‘indeterminate’ ratings (see Appendix E for detailed ratings). The quality of the evidence was graded from ‘very low’ to ‘high’ for this measurement property.

##### 3.4.1.3 Hypothesis testing (part of construct validity)

As none of the studies included an observer-rated measure of parent-child attachment, correlations had to meet the agreed cut-off against a self-report instrument only.

Twenty-five of 30 measures (83.3%) had studies reporting information for this measurement property. Of the prenatal measures, the *MFAS-23* and the *PFAS* were assigned ‘sufficient’ ratings, but the *MFAS-24, MFAS-20, PAI-21, MAAS-19* and *PAAS-19* were assigned ‘insufficient’ ratings, and the *MFAS-17* and the *PPBS* were assigned an ‘indeterminate’ rating. Of the postnatal measures, the *MABISC* and *MPAS* were assigned ‘sufficient’ ratings, the *MAI-26, PBQ-25, PBQ-14, MIBS-8* and *PPAS* were assigned ‘insufficient’ ratings, and the remaining measures were assigned ‘indeterminate’ ratings (i.e., when the hypothesis was not as defined by our review team). The quality of the evidence for all pre- and postnatal measures was graded ‘high’ for this measurement property.

##### 3.4.1.4 Cross-cultural validity (part of construct validity)

Although many studies (*n* = 38) aimed to adapt a given measure to different ethnic and language groups and some included back translation and other necessary procedures, none evaluated cross-cultural validity by comparing multiple groups by factor analysis and testing for differential item functioning (e.g., English- and Dutch-speaking), as stipulated in the COSMIN criteria. For this reason, this property is omitted from Table 5. See Appendix B for on overview of which measures have a version available in a different language.

##### 3.4.1.5 Measurement invariance (part of construct validity)

Only two measures (6.7%) could be rated for this measurement property (see Table 5). The *MPAS* and the *PPAS* were assigned ‘sufficient’ ratings of measurement invariance, demonstrating that these two measures appear to be measuring the same underlying construct (when tested with mothers and fathers, respectively). The quality of evidence for this property was ‘moderate’.

##### 3.4.1.6 Criterion validity

None of the studies reported on assessment of criterion validity. Hence, this property was omitted from Table 5.

#### 3.4.2 Assessment of reliability

##### 3.4.2.1 Internal consistency

Twenty-eight of the 30 measures (93.3%) had studies reporting on internal consistency. However, internal consistency could only be rated as ‘indeterminate’ for the majority of these measures because they did not demonstrate at least low evidence of ‘sufficient’ structural validity (as per the COSMIN criteria). Of the prenatal measures, the *PPBS* was assigned a ‘sufficient’ rating, whereas the *MFAS-17* was assigned an ‘insufficient’ rating; the remaining prenatal measures were assigned ‘indeterminate’ ratings. Of the postnatal measures, the *PBQ-22* was the only measure to receive a ‘sufficient’ rating. The *MIBS-J-7* was assigned an ‘insufficient’ rating, and the remaining measures were assigned ‘indeterminate’ ratings. The quality of the evidence was graded from ‘very low’ to ‘high’ for this measurement property (see Table 5).

##### 3.4.2.2 Reliability

Eleven of 30 measures (36.7%) had studies that reported test re-test reliability as defined by the COSMIN criteria. Of the maternal measures, the *PAI-21*, the *MPAS*, the *PBQ-25*, the *PBQ-14*, and the *MORS-SF* were all assigned ‘sufficient’ ratings, whereas the *MAI-31,* the *MABISC,* and the *MIBS-J* were assigned ‘insufficient’ ratings. Of the paternal measures, the *PFAS* was assigned a ‘sufficient’ rating whereas the *PPAS* was assigned an ‘insufficient’ rating. Evidence was graded from ‘very low’ to ‘moderate’ for this measurement property.

##### 3.4.2.3 Measurement error and responsiveness

None of the included studies measured measurement error and responsiveness as defined by the COSMIN criteria; hence, these could not be rated and were omitted from Table 5.

### 3.5 Inter-rater reliability

The agreement between the two reviewers was 89.0% (kappa = 0.85) for the risk of bias ratings, 79.7% (kappa = 0.68) for the measurement properties and 84.5% (kappa = 0.73) for quality of evidence (i.e., GRADE).

### 3.6 Clinimetrics/clinical utility

The clinimetrics or clinical utility of the 17 measures, presented in Table 3, were assessed in terms of time of administration, ease of scoring, Flesch Reading Ease (FRE), availability and conditions of use.

#### 3.6.1 Time to administer

Based on Bot et al.'s (2004) suggestion of a desirable completion time of less than 10 min, all 17 measures were assessed independently by two reviewers (SV and AM) and could be completed within 10 min. Most questionnaires (76.5%) took less than 2 min to complete. Four measures (the *MAAS, PAAS, MPAS* and *PPAS*) took longer than 2 min to administer because items covered multiple pages.

#### 3.6.2 Ease of scoring

In terms of ease of scoring, most measures (*n* = 14, 82.4%) received an easy rating due to their use of the Likert-scale scoring system. Response options ranged from four to seven options (see Table 3). Only two prenatal measures (the *MPAS* and the *PPAS*) received a moderate rating due to a combination of Likert-scale and simple formula scoring. The *MIRFS* is a two-part-scale, administered postnatally, in which the first sub-scale is rated as a Likert-scale and the second as a semantic differential scale; thus, it was judged to be difficult to score. As indicated in Table 3, in the majority of measures (*n* = 11, 64.7%) higher scores indicated stronger bonding or attachment, but in four (25%) postnatal measures higher scores were indicative of greater difficulties in the parent-reported bond with their infant. One scale (the *MORS-SF*) consisted of two sub-scales, whereby higher scores indicated higher maternal perceived levels of warmth as well as invasiveness. The ease of scores for one measure (the *MIRFS*) could not be reported because the scale development authors did not specify this in their paper (Thorstensson et al., 2012).

#### 3.6.3 Readability and comprehensiveness

Seventeen measures were assessed using the Flesch Reading Ease (FRE) test. As can be seen in Table 3, two measures (the *K-PAFAS* and the *PPBS*) received a poor rating. An excellent rating in terms of readability was given to one scale only (the *MIAS*). A fair rating was given to seven measures: *FAB, PAI-21, MAAS-19, PAAS, MAI, MPAS,* and *PPAS*, and seven measures, namely the *MFAS, PFAS, MABISC, PBQ-25, MIBS-8, MORS-SF,* and *MIRFS*, received a good rating. Of those measures, the *MFAS* and *PFAS* were the only antenatal measures.

#### 3.6.4 Availability and conditions of use

The majority of scales (*n* = 14, 82.4%) were easily accessible on the internet and free of charge; thus, receiving an excellent rating. Three scales, namely the *FAB*, the *PFAS* and *MIAS,* were difficult to obtain but free of charge; hence, they received a good rating.

## 4 Discussion

In this review we systematically examined the literature to identify, describe and evaluate the psychometric and clinimetric properties of self-report questionnaires for measuring the mother's or father's perception of their bond, attachment or relationship with their child. Seventeen original measures and their 13 modified versions, described in 65 articles from seven countries, were included and their methodological quality was carefully evaluated. Of these, a few measures were antenatal and postnatal measures for mothers (i.e., *MAAS, MFAS, MPAS*) or fathers (*PAAS, PFAS, PPAS*) only. The findings indicate that the evidence base for the robustness of self-report questionnaires measuring the parent-infant-relationship or bond is rather limited; consequently, we can only advise that these measures are used with some caution.

### 4.1 Considerations in relation to the COSMIN guidelines

The current 2018 COSMIN criteria appear to be the most stringent and complex to apply due to the multi-step process whereby firstly the quality of the measure development studies and the content validity studies were evaluated, secondly the methodological quality (risk of bias) of all studies was rated, thirdly the psychometric measurement properties were assessed and finally the quality of the evidence was graded. In other reviews of measures, reviewers either did not choose to apply the COSMIN criteria and opted to use other guidelines for rating each psychometric property (e.g., Lotzin et al., 2015; Perrelli et al., 2014), or they used an older COSMIN version (Terwee et al., 2007, in Wittkowski, Garrett, Calam, & Weisberg, 2017; Mokkink et al., 2010, in Bentley, Hartley, & Bucci, 2019, or De Vet, Terwee, Mokkink, & Knol, 2011, in Jewell et al., 2019).

Despite using the older COSMIN criteria, reviewers such as Jewell et al. (2019) have highlighted the arbitrary nature of cut-off scores which determine if a measurement property is ‘adequate’ or ‘inadequate’ because in some cases the statistical values indicative of a negative rating were very close to values suggesting a positive one. Furthermore, Jewell et al. (2019) critiqued the use of the ‘worst case counts’ rule because a single flaw in the study would result in only a ‘fair’ or even a negative rating which means that the adequacy and sufficiency of measurement properties and the methodological quality of any evidence are not necessarily a true reflection and most likely an underestimation. This criticism also fits with our observations when applying the COSMIN 2018 guidance.

In the application of the latest COSMIN guidance, we also became aware of how much practice and reporting standards have changed over the course of the last few decades; for example, the oldest measure our review identified was published in 1977 (e.g., the *FAB*). It was frustrating to note that authors reported some relevant information but did so without methodological consistency or rigor. For example, authors did not always report model fit statistics for confirmatory factor analyses so that they can be rated appropriately. Moreover, often authors only reported on correlation coefficients instead of reporting intraclass correlation coefficient (ICC) or kappa scores for (test-retest) reliability. This information is vital because the accurate assessment of a scale's structural validity depends on it.

When following the COSMIN 2018 criteria, we evaluated any modified versions of measures separately even if the total item count differed by only one item. However, in our findings we observed that psychometric testing was conducted less rigorously on refined or revised versions of measures compared to the originally developed measures. Thus, the risk of bias ratings, which show the methodological quality of each measure, might have been downgraded in line with the strict rules of COSMIN. This downgrading could be considered unfair given that some development work (e.g., pilot assessment or cognitive interviewing) might have been undertaken with the original scale.

Additionally, several of the included studies (e.g., Bienfait et al., 2011; Brockington et al., 2006, Brockington et al., 2001) tested for diagnostic accuracy (i.e., sensitivity and specificity of the measure in detecting bonding difficulties) and discriminant (or divergent) validity but these properties do not fall within the COSMIN taxonomy and consequently were not rated by us although we believe these to be important aspects of psychometric testing. Furthermore, only a few studies assessed aspects of interpretability (e.g., subgroup analyses, minimal important change, floor and ceiling effects, etc.) and thus, we could not report on these properties in our review.

### 4.2 Considerations relating to content validity

A measure's relevance, comprehensiveness and comprehensibility play a major deciding factor in why a measure may be chosen for clinical or research purposes; consequently, content validity may arguably be the most important psychometric property (Mokkink et al., 2018; Terwee et al., 2018). Based on COSMIN criteria, the methodological quality for the content validity of the original development measures identified in this review was ‘doubtful’ for the *MABISC, MPAS* and *PPAS* and ‘inadequate’ for the remaining 14 original measures. However, only 15 of the 65 included studies evaluated content validity at all, with the *MFAS* and *PBQ* having received the most attention, which was also noted in Tichelman et al.'s review (2019). Despite some studies evaluating ‘relevance’ and/or ‘comprehensibility’ among participants and/or professionals, none of the studies evaluated a measure's ‘comprehensiveness', highlighting the need for further research.

Following psychometric evaluation of all 17 measures, only three measures (*FAB, PAI-21,* and *MABISC*) were rated as ‘sufficient’ for overall content validity with the remaining measures receiving ‘inconsistent’ ratings. Nonetheless, the quality of the evidence regarding the *FAB* and the *PAI-21* was ‘very low’ and for the *MABISC* ‘low’ which indicates some uncertainty regarding the trustworthiness of the overall ratings.

Despite the increased trend towards paying more attention to evaluating content validity in terms of relevance, comprehensiveness and comprehensibility, particularly since 2010, there was a high percentage of studies being rated as ‘inadequate’ or ‘inconsistent’ with a ‘very low’ quality evidence. To mitigate against the very strict COSMIN criteria, we applied a more flexible approach by including studies that described other relevant aspects of content validity, such as appropriateness, suitability, acceptability, understandability, coherence, ambiguity and clarity of the items or overall measure. We consider these to be important aspects of content validity and they are potentially worthy of consideration as an expansion of content validity in the COSMIN guidelines. Although study authors described their evaluation of content validity, it often remained unclear what they had actually explored and how they had conducted the evaluation, because these studies were not developed or conducted in accordance with the high reporting standards of the COSMIN criteria. Hence, applying the COSMIN 2018 criteria resulted in most studies being rated as ‘doubtful’, ‘inadequate’, ‘inconsistent’ and/or ‘indeterminate’.

Furthermore, rating content validity according to the COSMIN criteria is a complex and multi-step process whereby the overall rating depends on the ratings of the development study, content validity study (or studies) and reviewers' ratings. On occasions when there were no content validity studies and the development studies received an ‘indeterminate’ rating, the overall ratings of the study were determined according to the reviewers' ratings which lead to increased subjectivity and to a higher likelihood of giving positive or ‘sufficient’ ratings. When content validation studies had been conducted, the measures tended to receive a lower overall score due to a lower quality of the content validity study. Thus, to minimise the bias and ambiguity, we encourage the reader to refer to individual ratings of the development study, content validity study (or studies) and reviewers' ratings which would give a more accurate overview of the measure's content validity.

### 4.3 Considerations relating to structural validity

The risk of bias for most studies assessing antenatal and postnatal measures was rated to be ‘adequate’ or ‘very good’. However, only two measures, namely the *PBQ-22* and the *MIBS-J-7* which were adapted versions of original measures, showed ‘sufficient’ evidence for structural validity and at least ‘moderate’ quality of evidence. The fact that many widely used measures had ‘insufficient’ evidence for structural validity is problematic and this issue needs to be explored further in future studies. In addition, most papers identified did not report the CFA estimation method used (e.g., Maximum Likelihood or Weighted Least Squares Mean and Variance), the appropriateness of which depends on several factors (see Rhemtulla, Brosseau-Liard, & Savalei, 2012 for recommendations). We suggest that future studies report this information to increase transparency and facilitate quality ratings.

Unlike Chiarotto, Ostelo, Boers, and Terwee (2018), who followed the COSMIN guidance strictly by only reporting the content validity and structural validity of the studies, we did report on other psychometric properties of the identified measures.

### 4.4 Considerations relating to construct validity

Construct validity comprises hypothesis testing, cross-cultural validity/measurement invariance and criterion validity; however, none of the included studies evaluated criterion validity. The risk of bias for hypothesis testing for antenatal and postnatal measures was mostly ‘very good’ with all studies consistently showing ‘high’ quality of evidence. Nevertheless, despite these promising results, only three antenatal measures (*MFAS-23, PFAS* and *K-PAFAS*) and two postnatal measures (*MABISC* and *MPAS*) offered the best evidence of any hypothesis testing undertaken. It is also important to note that construct validity was not assessed against any ‘gold standard’ self-report measure, since none has yet been identified. Furthermore, none of the studies included in this review used a ‘gold standard’ observer-rated measure of parent-child attachment to assess their measure's construct validity. This is clearly an area of further investigation.

Measurement invariance was rarely assessed in the identified measures. Based on ‘adequate’ methodological quality (i.e., risk of bias ratings), the *MPAS* and the paternal equivalent, the *PPAS*, were assigned ‘sufficient’ ratings with ‘moderate’ quality of evidence.

Due to little evidence of construct validity, more research needs to be undertaken.

### 4.5 Considerations relating to reliability

Except for three antenatal versions (*FAB*, *MAAS-13* and *PAAS-13*) and three postnatal versions (*MABISC, PBQ-16* and *MIAS*), the quality of evidence for the internal consistency of ten antenatal versions and 13 postnatal versions was considered to be ‘high’. However, most studies did not provide enough information about the internal consistency of the antenatal and postnatal measures assessed and only the *PBQ* showed ‘sufficient’ evidence for internal consistency. This is because internal consistency could only be rated if the studies demonstrated at least low evidence of ‘sufficient’ structural validity (as per the 2018 COSMIN criteria); if a measure does not demonstrate good structural validity, there is no confidence that those subscales exist. This explains why most of the measures received ‘indeterminate’ ratings. In addition, all of the studies reported internal consistency as Cronbach's Alpha values. Although this approach is the most popular and widely applied statistic of internal consistency (Dunn et al., 2014), it has been criticised for having several flaws. For example, it is considered an inappropriate statistic to estimate a scale's reliability (Peters, 2014) and homogeneity of unidimensionality (Green, Lissitz, & Mulaik, 1977; Schmitt, 1996; Sijtsma, 2009). Thus, researchers evaluating the internal consistency of measures are encouraged to use alternatives, such as McDonald's omega in the future (Dunn et al., 2013; Peters, 2014).

The reliability of measures could be assessed for two antenatal measures only because information for the remaining antenatal measures was not available. Whilst the reliability for the *PAI-21* was ‘sufficient’ with ‘moderate’ quality of evidence, the reliability for the *K-PAFAS* was ‘insufficient’ with ‘low’ quality of evidence. Thus, based on the available evidence, the *PAI-21* appears to be the most robust antenatal measures to be used with pregnant women.

In relation to the 17 versions of postnatal measures, seven versions (i.e., *MAI-26, MPAS, PBQ-25, PBQ-14, MIBS-J-10, MORS-SF* and *PPAS*) evaluated the methodological quality (i.e., risk of bias ratings) of reliability in ten studies which varied between ‘adequate’, ‘doubtful’ and ‘inadequate’ ratings. However, conclusive summaries regarding the methodological quality cannot be provided due to these measures presenting with mixed evidence, depending on the country, language and sample size of each conducted study. Of all postnatal measures, only two measures were considered to have ‘sufficient’ evidence for their reliability with ‘moderate’ quality of evidence. Hence, the *MPAS* and the *PBQ-25* appear to be the most reliable postnatal measures.

However, it is important to note that none of the identified studies reported measurement error and responsiveness as part of their assessment of a scale's additional reliability. Clearly, more work is needed to fully establish the reliability of these scales.

### 4.6 Considerations relating to clinimetric properties

The assessment of administrative properties of a measure in addition to its psychometric properties has been recommended (e.g., Thornicroft & Slade, 2000; Wittkowski et al., 2017). As it is assumed that their modified and often simplified versions would achieve similar ratings, the clinimetric properties were assessed for the original 17 measures only. Except for three measures (e.g., *MPAS, PPAS* and *MIRFS*), the scoring of all measures was rated to be ‘easy’. In addition, none of the measures had an excessive number of items. Hence their completion time was rated as ‘excellent’. With item numbers ranging from seven (e.g., the *MIBS-7*) to 26 (e.g., the *MAI*), all measures could be completed under five minutes, which suggests that they are acceptable and feasible measures, suitable for the use in routine outcome assessments.

In terms of readability and comprehension, all measures (except for the *K-PAFAS*) obtained ratings in the ‘fair’ to ‘excellent’ range. Of the antenatal measures, the *MFAS* obtained the best (i.e., ‘good’) score in readability, whereas six of the nine postnatal measures were rated to be ‘good’ or ‘excellent’ and hence easy to understand (e.g., *MABISC, PBQ-25, MIBS-8, MORS-SF, MIRFS* and *MIAS*). Hereby it is noteworthy that the item exploring whether the women or their partners felt that the woman's body was ‘ugly’ (reverse scored) in the *MFAS* (and the paternal equivalent, the *PFAS)* is occasionally removed from the scale because it does not refer to maternal feelings (Müller & Ferketich, 1993; Van den Bergh and Simons, 2009). With the Flesch readability scores ranging from 55.6 (*K-PAFAS*) to 96.0 (*MIAS*), all of the measures appeared to be acceptable.

### 4.7 Strengths and limitations of the review

A clear strength of this review is its comprehensiveness evidenced by the fact that eight databases were searched and more than 12,000 records were screened, in all languages and publication years. Any measure and study inclusion criteria were determined in advance and registered. In addition, data extraction and evaluation processes were verified by an independent rater and these showed good to excellent inter-rater agreement and reliability. Furthermore, by reporting information pertaining to the clinimetric properties of the identified measures, we assist clinicians and researchers in their assessment of how ‘user-friendly’ a measure is. Furthermore, the strict application of the latest COSMIN criteria (2018) ensured that a rigorous assessment was undertaken of the validity and reliability evidence of the identified measures. Compared to previous reviews in this area (e.g., Perrelli et al., 2014), the use of the most recent and stringent COSMIN criteria adds substantial credibility to this detailed assessment of measures. Finally, although the methodological quality of studies varied, we chose to report those variations rather than exclude those studies.

In terms of limitations, it should be acknowledged that the search was restricted to peer-reviewed studies only, which introduces a publication bias (Rothstein, 2014). However, although unpublished measures with good psychometric properties may exist, without being published appropriately their impact in the field may be minimal. For this reason, Lotzin et al. (2015) searched all available literature in their review of observational tools for measuring parent-infant-interaction, but then decided to exclud tools that were only used in one or no peer-reviewed journal articles.

Although we included different language versions of identified measures in our evidence synthesis (e.g., *K-PAFAS*, *MIRFS* and *MIAS*), it proved impossible for us to apply the COSMIN criteria to assess the psychometric properties of measures in identified studies if they were not written in English. However, for comprehensiveness we offer further information on those studies, including their sample size and the psychometric properties tested.

In addition, when rating the content validity of studies, we chose to deviate from the stricter 2018 COSMIN guidelines on four occasions. Firstly, we opted to rate the psychometric properties of all studies evaluating content validity even if the study's methodological quality (i.e., risk of bias) was ‘inadequate’ as this resulted in a detailed and thorough overview of all included measures. Secondly, we adapted the criteria of rating the risk of bias of the measure development studies slightly in cases where the authors had presented ‘adequate’ evidence regarding the study's conduct; however, this could have resulted in assigning higher risk of bias ratings in some cases. Thirdly, we modified the criteria for structural validity since many studies in this review had undertaken EFA but the COSMIN criteria do not include guidance on how to rate the results of an EFA. Fourthly, we deviated from the guidelines when rating hypothesis testing and the results of this measurement property should be interpreted with caution.

Another limitation that should be acknowledged is the fact that we excluded measures in which the parent-infant-relationship was examined but only alongside other and arguably less relevant aspects, such as exploring the woman's body or diet. For example, despite containing seven items that could be said to reflect the mother's attitude towards the developing fetus, the 60-item *Maternal Adjustment and Maternal Attitude (MAMA)* questionnaire (Kumar et al., 1984) was excluded because it contained many other items relating to the mother's perceptions of her body or items of somatic symptoms, the marital relationship, attitudes to sex and attitudes towards pregnancy. The postnatal version of the *MAMA* was also excluded, although it was judged to contain slightly more relevant items (*n* = 9). Only 13 of the 26-item *What Being The Parent of a Baby is Like (WBTPBL*, Pridham & Chang, 1985) scale related directly to the parent-infant-relationship with the other items asking about the new parent's adaptation to parenthood, relationships with family members and the stress of being a new parent. Finally, although *the Maternal Infant Responsiveness Instrument (MIRI;* Amankwaa, Younger, Best, & Pickler, 2002) partly met our inclusion criteria, the scale mostly examines parental perceptions of baby responsiveness and was therefore excluded.

### 4.8 Implications for research and practice

The fact that we identified a lack of evidence for robust psychometric properties across a wide variety of antenatal and postnatal parent-report measures is problematic because any conclusions based on these measures will have inherent limitations.

Although some of these measures may have been extensively used (e.g., the *PBQ*) or their use (e.g., *the PBQ and the MORS-SF)* may have been recommended (Royal College of Psychiatrists, 2018), it is advisable that clinicians and researchers alike scrutinise each measure in order to determine if it fits their purpose. For example, all of these measures were validated using predominantly non-clinical populations (with the exception of the *PBQ-25* and the *MABISC*) and this means that clinicians and researchers need to consider a measure's relevance when applied to their intended population or purpose. Besides, in line with the review's aims, we only included studies specifically evaluating psychometric properties but we are aware that studies may exist that report on a measure's use with clinical populations (e.g., see Wittkowski et al., 2010) and possibly on correlations with observer-rated measures as well. The reader is advised that we did not search for those studies or indeed included them in this review. Given the recent proliferation of measures being adapted for use in other countries and in languages other than English, we believe that there is a need for appropriate and more stringent testing for cross-cultural validity. For example, studies with different cultural or ethnic groups should conduct factor analyses for multiple groups (e.g., in English and in Dutch) and complete measurements of invariance or differential item functioning (DIF) to provide information on whether the measures are comparable when used in differing cultural contexts. This could be one of the future directions when testing psychometric properties of the measures.

We also believe that future studies conducting content validity evaluation should describe more explicitly how they evaluated content validity and what aspects they did evaluate and to consult and follow COSMIN criteria when developing the method of a new measure or assessing the method of an already existing measure. This may include conducting a qualitative study (i.e., a focus group or interviews), using appropriate data collection and analysis methods and ideally exploring the relevance, comprehensiveness and comprehensibility of the measure among a sufficient sample of participants and professionals, which would lead to a higher quality and more credible evidence of the measure's content validity.

## 5 Conclusion

This is the first systematic review to provide a synthesis of robust validity and reliability evidence for available self-report measures of the parent-infant-relationship. A total of 17 measures and 13 modified versions were identified and evaluated, of which the majority lacked adequate methodological quality despite being widely used and with some being recommended measures. Only the *Postpartum Bonding Questionnaire* (PBQ), and some of its modified versions, were found to demonstrate sufficient evidence for structural validity, internal consistency and reliability with high quality of evidence. The PBQ was also the most frequently adapted tool which is indicative of its perceived relevance and popularity in this field. However, due to the inadequate methodological quality and insufficient psychometric measurement evaluation of most measures, in addition to the lack of comprehensive psychometric evaluation of many measures, firm conclusions regarding the most valid and reliable parent-infant-relationship measure(s) cannot be drawn.

The current review is important and timely given the increasing importance of routine self-report outcome monitoring within a range of perinatal services and within research studies (e.g., NHS England, NHS Improvement, and National Collaborating Centre for Mental Health, 2018). Despite the wealth of antenatal and postnatal measures, the psychometric properties of these tools remain poor and understudied. It is advisable that future researchers developing new or modified measures follow the current COSMIN guidelines and that research into evaluation of psychometric properties would continue in order to bring measures to the industry standard and facilitate the selection of the most robust antenatal and postnatal measures by researchers and clinicians.

## Funding

This review was undertaken as part of the THRIVE project funded by the National Institute for Health Research (NIHR) Public Health Research Programme (PHR Project: 11/3002/01), led by MH. As part of this grant, CG, AM and SV were employed as researchers and AW was their supervisor as well as Manchester lead for the THRIVE project. The funder had no direct involvement in the conduct of this review. The views expressed are those of the authors and not necessarily those of the NHS, the NIHR or the Department of Health and Social Care. MH was supported by the MRC/10.13039/501100000589CSO Quinquennial funding of the Relationships and Health Improvement Programme, which is part of the Social and Public Health Sciences Unit, based at 10.13039/501100000853University of Glasgow, MC_UU_12017-11 and SPHSU11.

## Contributions

AW devised the idea for this review and oversaw the whole review process. CG and AW wrote the initial protocol which was later updated with SV and AM's assistance. SV, CG and AM conducted the literature searches. AM and SV rated the included studies, under AW's supervision. Any disagreements were resolved through consultation with the fifth reviewer, MH. All authors contributed to drafts and approved the final manuscript.

## Declaration of Competing Interest

All authors declare that they have no conflict of interest.

## Figures and Tables

**Table:** 

Measure(s) evaluated	Authors	Language	Sample size	Psychometric properties tested
MFAS	Lauriola et al. (2010)	Italian	254 women	Internal reliability; construct validity
MFAS	Andrek et al. (2016)	Hungarian	114 women	Internal reliability
PAI	Jurgens et al. (2009)	French	112 women	Internal reliability; construct validity
PAI	Lauriola et al. (2010)	Italian	254 women	Internal reliability; construct validity
PAI	Pavše et al. (2019)	Slovenian	619 women	Internal reliability; structural validity
MAAS	Camarneiro & Justo (2010)	Portuguese	212 couples	Internal reliability; structural validity
MAAS	Lauriola et al. (2010)	Italian	254 women	Internal reliability; construct validity
MAAS	Denis et al. (2013)	French	117 women	Internal reliability; construct validity; concurrent validity; divergent validity
MAAS	Nie & Fan (2017)	Chinese	545 women	Internal reliability; convergent validity
PAAS	Camarneiro & Justo (2010)	Portuguese	212 couples	Internal reliability; structural validity
MAI	Kavlak & Sirin, 2009	Turkish	165 women	Internal reliability; content validity
PBQ	Yalcin et al. (2014)	Turkish	189 women	Internal reliability
MIBS	Figueiredo et al. (2005)	Portuguese	456 parents	Internal reliability; test-retest reliability
MIBS	Yalcin et al. (2014)	Turkish	189 women	Internal reliability
MORS-SF	Danis et al. (2012)	Hungarian	1164 parents	Internal reliability

**Table:** 

	RELEVANCE					COMPREHENSIVENESS					COMPREHENSIBILITY					TOTAL CONTENT VALIDITY
	Outcome measure development study	Content validity studies	Reviewers ratings	Ratings of results	Quality of evidence	Outcome measure development study	Content validity studies	Reviewers ratings	Ratings of results	Quality of evidence	Outcome measure development study	Content validity studies	Reviewers ratings	Ratings of results	Quality of evidence	
FAB	[?]	None	[+; +]	[+]	VERY LOW	[?]	None	[+; +]	[+]	VERY LOW	[?]	None	[+; +]	[+]	VERY LOW	**[+]**
MFAS-24	[?]	[±; ±]	[+; +]	[±]	VERY LOW	[?]	[−; −]	[+; +]	[±]	VERY LOW	[?]	[?;?]	[+; ±]	[±]	VERY LOW	**[±]**
MFAS-20	NA	[±]	[+; +]	[±]	VERY LOW	NA	[−]	[+; +]	[±]	VERY LOW	NA	[?]	[+; ±]	[±]	VERY LOW	**[±]**
PAI	[?]	[+]	[+; +]	[+]	VERY LOW	[?]	[+]	[+; +]	[+]	VERY LOW	[?]	[?]	[+; +]	[+]	VERY LOW	**[+]**
MAAS	[+]	[±]	[+; +]	[±]	VERY LOW	[+]	[+]	[+; +]	[+]	VERY LOW	[+]	[?]	[+; +]	[+]	VERY LOW	**[±]**
PPBS	[+]	None	[+; +]	[+]	VERY LOW	[−]	None	[+; −]	[±]	VERY LOW	[−]	None	[+; +]	[±]	VERY LOW	[±]
PFAS	[?]	None	[+; +]	[+]	VERY LOW	[+]	None	[+; +]	[+]	VERY LOW	[?]	None	[+; ±]	[±]	VERY LOW	[±]
PAAS	[+]	[−]	[+; +]	[±]	VERY LOW	[+]	[−]	[+; +]	[±]	VERY LOW	[?]	[?]	[+; +]	[+]	VERY LOW	**[±]**
K-PAFAS	[+]	[±]	[+; ±]	[±]	VERY LOW	[+]	[−]	[+; +]	[±]	VERY LOW	[?]	[−]	[+; ±]	[±]	VERY LOW	**[±]**
MAI	[?]	[±; ±]	[+; +]	[±]	VERY LOW	[+]	[+; +]	[+; +]	[+]	VERY LOW	[?]	[?;?]	[+; +]	[+]	VERY LOW	**[±]**
MIAS	[?]	None	[+; ±]	[±]	VERY LOW	[+]	None	[+; +]	[+]	VERY LOW	[?]	None	[+; ±]	[±]	VERY LOW	**[±]**
MABISC	[+]	None	[+; +]	[+]	LOW	[+]	None	[+; +]	[+]	LOW	[+]	None	[+; +]	[+]	LOW	**[+]**
MPAS	[+]	[±]	[+; +]	[±]	LOW	[+]	[−]	[+; +]	[±]	LOW	[?]	[−]	[+; +]	[±]	LOW	**[±]**
PBQ-25	[±]	[+; +]	[+; +]	[+]	VERY LOW	[−]	[+; −]	[+; +]	[±]	VERY LOW	[?]	[+; +]	[+; +]	[+]	VERY LOW	**[±]**
PBQ-19	NA	[−]	[+; +]	[±]	VERY LOW	NA	[−]	[+; +]	[±]	VERY LOW	NA	[?]	[+; +]	[+]	VERY LOW	**[±]**
MIBS	[±]	None	[+; +]	[+]	VERY LOW	[−]	None	[+; ±]	[±]	VERY LOW	[?]	None	[+; +]	[+]	VERY LOW	**[±]**
MIRFS	[?]	[±; ±]	[+; ±]	[±]	VERY LOW	[+]	[−; −]	[+; ±]	[±]	VERY LOW	[?]	[?; +]	[+; ±]	[±]	VERY LOW	**[±]**
MORS-SF	[?]	None	[+; +]	[+]	VERY LOW	[−]	None	[+; +]	[±]	VERY LOW	[?]	None	[+; +]	[+]	VERY LOW	**[±]**
PPAS	[+]	[±]	[+; +]	[±]	LOW	[+]	[−]	[+; +]	[±]	LOW	[?]	[−]	[+; +]	[±]	LOW	**[±]**

*Notes.* [+] = sufficient. [?] = indeterminate. [±] = inconsistent. [−] = insufficient. NA—not applicable. None = no content validity studies conducted. The multiple ratings per box indicate either multiple content validity studies or multiple reviewers' ratings.

**Table:** 

**#**	Type1	Type2	Measure	Language of scale	Paper (by first author and year)	*n*	Structural validity	*n*	Internal consistency	*n*	Reliability	*n*	Hypothesis testing	*n*	Measurement invariance
1	Pre	Mat	**FAB**	English	Leifer, 1977	–	–	–	–	–	–	–	–	–	–
2	Pre	Mat	**MFAS-24**	English	Cranley, 1981	–	–	71	Very good [?]	–	–	71	Inadequate [−]	–	–
3	Pre	Mat		Tamil	Lingeswaran, 2012	–	–	230	Inadequate [?]	–	–	–	Very good [?]	–	–
4	Pre	Mat		Hungarian	Andrek, 2016	114	Inadequate [?]	114	Very good [?]	–	–	–	Inadequate [?]	–	–
5	Pre	Mat		German	Doster, 2018	324	Adequate [−]	324	Very good [?]	–	–	324	Very good [−]	–	–
6	Pre	Mat	MFAS-23	English	Müller & Ferketich, 1993	681	Adequate [−]	681	Very good [?]	–	–	–	–	–	–
7	Pre	Mat		English	Müller, 1993	336	Adequate [?]	336	Very good [?]	–	–	336	Very good [+]	–	–
8	Pre	Mat	MFAS-20	Italian	Busonera, 2016a	482	Very good [−]	482	Very good [?]	–	–	482	Very good [−]	–	–
9	Pre	Mat	MFAS-17	Swedish	Seimyr, 2009	298	Adequate [?]	298	Very good [?]	–	–	–	Very good [?]	–	–
10	Pre	Mat		Swedish	Sjögren, 2004	76	Inadequate [+]	–	–	–	–	76	Very good [?]	–	–
11	Pre	Mat	**PAI-21**	English	Müller, 1993^1^	336	Adequate [−]	336	Very good [?]	–	–	336	Adequate [−]	–	–
12	Pre	Mat		English	Müller, 1996	–	–	196	Very good [?]	–	–	196	Very good [−]	–	–
13	Pre	Mat		English	Gau, 2003	349	Very good [−]	349	Very good [?]	–	–	–	–	–	–
14	Pre	Mat		Swedish	Pallant, 2014	775	Very good [−]	775	Very good [?]	–	–	–	–	–	–
15	Pre	Mat		Italian	Barone, 2014	130	Adequate [+]	130	Very good [?]	–	–	–	Very good [?]	–	–
16	Pre	Mat		Italian	Busonera, 2017a	535	Very good [−]	535	Very good [?]	–	–	535	Very good [−]	–	–
17	Pre	Mat		Italian	Della Vedova, 2008	214	Adequate [−]	214	Very good [?]	–	–	–	Very good [?]	–	–
18	Pre	Mat		Persian	Samani, 2016	322	Very good [−]	322	Very good [?]	322	Adequate [+]	–	Very good [?]	–	–
19	Pre	Mat	**MAAS-19**	English	Condon, 1993	150	Adequate [−]	150	Inadequate [?]	–	–	–	–	–	–
20	Pre	Mat		English	Condon, 1997	–	–	–	–	–	–	–	Very good [?]	–	–
21	Pre	Mat		Italian	Busonera, 2016b	482	Very good [−]	482	Very good [?]	–	–	482	Very good [−]	–	–
22	Pre	Mat		Spanish	Navarro-Aresti, 2016	–	–	525	Very good [?]	–	–	–	–	–	–
23	Pre	Mat		Turkish	Golbasi, 2015	190	Adequate [?]	190	Very good [?]		–		–	–	–
24	Pre	Mat		Hungarian	Mako Deak, 2014	–	–	237	Very good [?]	–	–	237	Very good [+]	–	–
25	Pre	Mat		Dutch	Van Bussel, 2010b	–	–	403	Very good [?]	–	–	–	Inadequate [?]	–	–
26	Pre	Mat	MAAS-13	German	Göbel, 2019	263	Adequate [−]	263	Very good [?]	–	–	–	–	–	–
27	Pre	Mat	MAAS-12	Spanish	Navarro-Aresti, 2016	525	Very good [−]	525	Very good [?]	–	–	–	–	–	–
28	Pre	Mat	**PPBS**	Dutch	Cuijlits, 2016	1050	Very good [+]	529	Very good [+]	–	–	1050	Very good [?]	–	–
29	Pre	Pat	**PFAS**	English	Weaver, 1983	–	–	100	Inadequate [?]	–	–	100	Inadequate [?]	–	–
30	Pre	Pat		Swedish	Seimyr, 2009	298	Adequate [?]	298	Very good [?]	–	–	298	Very good [?]	–	–
31	Pre	Pat	**PAAS-19**	English	Condon, 1993	112	Doubtful [−]	112	Inadequate [?]	–	–	–	–	–	–
32	Pre	Pat		English	Condon, 2013	–	–	–	–	–	–	204	Very good [−]	–	–
33	Pre	Pat		Italian	Della Vedova, 2017	65	Doubtful [−]	65	Very good [?]	–	–	65	Very good [−]	–	–
34	Pre	Pat	PAAS-13	German	Göbel, 2019	128	Adequate [−]	128	Very good [?]	–	–	–	–	–	–
35	Pre	Pat	**K-PAFAS**	Korean	Noh, 2017	200	Very good [−]	200	Very good [?]	200	Doubtful [+]	200	Very good [+]	–	–
36	Post	Mat	**MAI-26**	English	Müller, 1994^2^	–	–	196	Very good [?]	148	Doubtful [+]	196	Adequate [−]	–	–
37	Post	Mat		English	Müller, 1996	–	–	196	Very good [?]	–	–	196	Very good [−]	–	–
38	Post	Mat		Korean	Shin, 2007	196	Adequate [+]	196	Very good [?]	–	–	196	Very good [−]	–	–
39	Post	Mat		Chinese	Chen, 2013	181	Adequate [+]	181	Very good [?]	–	–	181	Very good [−]	–	–
40	Post	Mat	**MIAS**	Hindi	Bhakoo, 1994	100	Inadequate [?]	100	Adequate [?]	–	–	–	Very good [?]	–	–
41	Post	Mat	**MABISC**	English	Hackney, 1996	–	–	–	–	–	–	–	Inadequate [?]	–	–
42	Post	Mat		Norwegian	Hoivik, 2013	76	Doubtful [+]	76	Doubtful [?]	–	–	76	Very good [+]	–	–
43	Post	Mat	**MPAS**	English	Condon, 1998	212	Adequate [−]	212	Very good [?]	212	Adequate [+]	212	Very good [+]	–	–
44	Post	Mat		English	Feldstein, 2004	–	–	59	Very good [?]	–	–	59	Very good [−]	–	–
45	Post	Mat		Italian	Scopesi, 2004	210	Very good								
[−]	210	Very good [?]	–	Inadequate [−]	–	Very good [?]	–	–							
46	Post	Mat		Dutch	Van Bussel, 2010a	–	–	–	–	–	–	263	Very good [+]	–	–
47	Post	Mat		Spanish	Riera-Martin, 2016	571	Very good [−]	571	Very good [?]	–	–	–	Very good [?]	571	Adequate [+]
48	Post	Mat	**PBQ-25**	English	Brockington, 2001	104	Adequate [+]	–	–	104	Inadequate [+]	–	–	–	–
49	Post	Mat		English	Brockington, 2006	–	–	–	–	–	–	–	–	–	–
50	Post	Mat		German	Reck, 2006	862	Adequate [−]	862	Very good [?]	–	–	–	Very good [?]	–	–
51	Post	Mat		English	Wittkowski, 2007	–	–	96	Very good [?]	–	–	96	Very good [−]	–	–
52	Post	Mat		English	Wittkowski, 2010	132	Adequate [−]	–	–	–	–	–	–	–	–
53	Post	Mat		Chinese	Siu, 2010	–	–	–	–	–	–	–	–	–	–
54	Post	Mat		Dutch	Van Bussel, 2010a	–	–	263	Very good [?]	–	–	263	Very good [−]	–	–
55	Post	Mat		Japanese	Kaneko, 2014	1786	Doubtful [?]	1786	Very good [?]	–	–	–	Very good [?]	–	–
56	Post	Mat		Japanese	Suetsugu, 2015	199	Adequate [?]	199	Very good [?]	199	Doubtful [+]	199	Very good [+]	–	–
57	Post	Mat		Spanish	Garcia-Esteve, 2016	840	Very good [−]	–	–	–	–	–	–	–	–
58	Post	Mat		Portuguese (Brazil)	Baldisserotto, 2018	–	–	–	–	–	–	–	–	–	–
59	Post	Mat		Italian	Busonera, 2017b	123	Adequate [+]	123	Very good [?]	–	–	123	Very good [−]	–	–
60	Post	Mat		Japanese	Ohashi, 2016	364	Very good [−]	364	Very good [?]	364	Adequate [+]	–	Very good [?]	–	–
61	Post	Mat	PBQ-16	German	Reck, 2006	–	–	862	Inadequate [?]	–	–	–	Very good [?]	–	–
62	Post	Mat	PBQ-22	English	Wittkowski, 2010	132	Adequate [+]	132	Very good [+]	–	–	–	Very good [?]	–	–
63	Post	Mat	PBQ-16-J^3^	Japanese	Kaneko, 2014	1786	Doubtful [?]	1786	Very good [?]	–	–	–	Very good [?]	–	–
64	Post	Mat	PBQ-14	Japanese	Suetsugu, 2015	199	Adequate [?]	199	Very good [?]	199	Doubtful [+]	199	Very good [−]	–	–
65	Post	Mat	PBQ-19^4^	Tamil	Vengadavaradan, 2019	250	Adequate [?]	–	–	–	–	–	Very good [?]	–	–
66	Post	Mat	S-PBQ	English	Kinsey, 2014	3005	Adequate [?]	3005	Very good [?]	–	–	–	Very good [?]	–	–
67	Post	Mat	**MIBS-8**	English	Taylor, 2005	162	Adequate [?]	162	Very good [?]	–	–	–	–	–	–
68	Post	Mat		English	Wittkowski, 2007	–	–	96	Very good [?]	–	–	96	Very good [−]	–	–
69	Post	Mat		Dutch	Van Bussel, 2010a	–	–	263	Very good [?]	–	–	263	Very good [−]	–	–
70	Post	Mat		French	Bienfait, 2011	–	–	–	–	–	–	78	Very good [+]	–	–
71	Post	Mat	MIBS-J-10	Japanese	Yoshida, 2012	554	Very good [−]	554	Very good [?]	554	Doubtful [−]	–	Very good [?]	–	–
72	Post	Mat	MIBS-J-7	Japanese	Ohara, 2016	375	Very good [+]	751	Very good [−]	–	–	–	Very good [?]	–	–
73	Post	Mat	**MIRFS**	Swedish	Thorstensson, Nissen 2012b	395	Adequate [?]	395	Very good [?]	–	–	–	–	–	–
74	Post	Mat		Swedish	Thorstensson, Hertfelt Wahn, 2012a	–	–	–	–	–	–	–	–	–	–
75	Post	Mat	**MORS-SF**^5^	English	Oates, 2018	–	–	100	Very good [?]	–	–	–	Very good [?]	–	–
76	Post	Mat		Hungarian	Oates, 2018	–	–	331	Very good [?]	–	–	–	Very good [?]	–	–
77	Post	Mat		English	Oates, 2019	100	Inadequate [−]	–	–	–	–	–	–	–	–
78	Post	Mat		Hungarian	Oates, 2019	134	Inadequate [−]	–	–	36	Doubtful [+]	–	–	–	–
79	Post	Mat		English and Hungarian	Oates, 2019	243	Adequate [−]	243	Very good [?]	–	–	–	–	–	–
80	Post	Pat	**PPAS**	English	Condon, 2008	461	Adequate [−]	461	Very good [?]	–	–	–	Very good [?]	–	–
81	Post	Pat		English	Feldstein, 2004	–	–	38	Very good [?]	–	–	–	Very good [?]	–	–
82	Post	Pat		English	Condon, 2013	–	–	–	–	204	Inadequate [−]	204	Very good [−]	–	–
83	Post	Pat		Spanish	Riera-Martin, 2016	376	Very good [−]	376	Very good [?]	–	–	–	Very good [?]	376	Adequate [+]

*Notes.* [+] = sufficient. [−] = insufficient. [?] = indeterminate. [±] = inconsistent. Structural validity ratings were based on the best fitting model presented in the paper (this was not necessarily the factor structure proposed by the original authors). As per the COSMIN criteria, internal consistency could only be rated as sufficient if there was at least low evidence of sufficient structural validity (otherwise an indeterminate rating was assigned). Some papers are listed more than once as the authors tested psychometric properties of more than one measure.

1 EFA was conducted on 29-item PAI, resulting in a 21-item scale.

2 EFA was conducted on a 31-item MAI, resulting in a 26-item scale.

3 PBQ-J-16 contains different items from the PBQ-16.

4 EFA conducted on full PBQ, resulting in 19-item scale.

5 MORS-SF EFA conducted on 44-items, resulting in 14-item scale.

## Appendix A Example search strategy in OVID

1. ((parent* or maternal or paternal or mother* or father*) adj7 (child or infant or newborn or foet* or fetus or fetal or baby or neonate)).mp

2. (antenat* or prenat* or puerper* or postnat* or postpart* or peripartum or pregnan* or perinat*).mp

3. ((measure* or scale$ or questionnaire$ or construct$ or tool$ or inventor* or instrument$ or test*) adj7 (attachment or relation* or bond* or orientation or synchrony or synchronicity or “emotional availability” or attitude* or belief* or responsive* or feel* or interact*)).mp

4. 1 and 2 and 3

5. limit 4 to all journals

6. limit 5 to (female or humans or male).

7. limit 6 to peer reviewed journal.

8. limit 7 to original articles.

9. limit 8 to (2200 psychometrics & statistics & methodology or 2220 tests & testing or 2222 developmental scales & schedules or 2223 personality scales & inventories or 2224 clinical psychological testing or 2225 neuropsychological assessment or 2226 health psychology testing or 2240 statistics & mathematics or 2260 research methods & experimental design or 2520 neuropsychology & neurology or 2600 psychology & the humanities or 2840 psychosocial & personality development or 3000 social psychology or 3040 social perception & cognition or 3360 health psychology & medicine).

## Appendix B Overview and reference list of non-English studies identified in the systematic search

### B.1 Reference list for Appendix B

Andrek, A.., Hadhazi, E., & Kekecz, Z. (2016). Az anya-magzat kötődés mérő Mother Fetus Attachment Scale kérdőív magyar nyelvű adaptálása és felhasználásának lehetőségei az ultrahang-kommunikációs vizsgálatok során. [The Hungarian adaptation and potential use of the Mother Fetus Attachment Scale questionnaire measuring mother-to-fetus attachment during ultrasound communication examinations]. *Orvosi Hetilap, 157(20)*, 789–795.

Camarneiro, A. P., & Justo, J. (2010). Padrões de vinculação pré-natal. Contributos para a adaptação da Maternal and Paternal Antenatal Attachment Scale em casais durante o segundo trimestre de gestação na região Centro de Portugal [Contributions to the adaptation of the Maternal and Paternal Antenatal Attachment Scale in couples during the second trimester of gestation in the central region of Portugal]. *Revista Portuguesa de Pedopsiquiatria, 28,* 7–22.

Danis. I., Scheuring, N., Gervai, J., Oats, J. M., & Czinner, A. (2012). A röviditett Szulö-Csecsemö Kapcsolat Skála magyar valtozátnak (H-MORS-SF) pszichometriai mutatói nagy mintán. [Psychometric parameters of the Hungarian version of Mothers' Object Relations Scales - Short Form (H-MORS-SF) in a large sample]. *Psychiatria Hungarica: A Magyar Pszichiátriai Társaság tudományos folyóirata, 27(6),* 392–405.

Denis, A., Callahan, S., & Bouvard, M. (2013). Examen des propriétés psychométriques de la traduction française de la Maternal Antenatal Attachment Scale (MAAS). [Examination of the psychometric properties of the French translation of the Maternal Antenatal Attachment Scale (MAAS)]. *L’Encephale, 41(1),* 32–38.

Figueiredo, B., Marques, A., Costa, R., Pacheco, A., & Pais, A. (2005). Bonding: Escala para avaliar o envolvimento emotional dos pais com o bebé [Bonding: Scale to evaluate parents' emotional involvement with their infant]. *Psychologica, 40,* 133–154.

Jurgens, M. A., Levy-Rueff, M., Goffinet, F., Golse, B., & Beauquier-Macotta, B. (2009). Étude des propriétés psychométriques d’une echelle d’attachement prénatal. Version franc¸aise de la Prenatal Attachment Inventory (PAI, Müller, 1993). [Psychometric properties of the French version of the prenatal attachment inventory in 112 pregnant women]. *L’Encéphale, 36(3),* 219–225.

Kavlak, O., & Sirin, A. (2009). The Turkish version of Maternal Attachment Inventory. *Journal of Human Sciences, 6(1)*, 188–202.

Lauriola, M., Panno, A., Riccardi, C., & Tagliatela, D. (2010). La misura dell’attaccamento materno prenatale: un confronto psicometrico di tre strumenti di valutazione. [The measure of maternal prenatal attachment: a psychometric comparison of three assessment tools]. *Infanzia e adolescenza, 9(3)*, 135–150.

Nie, G., & Fan, H.-X. (2017). Validity and reliability of the Chinese version Maternal Antenatal Attachment Scale. *Chinese Journal of Clinical Psychology, 4*, 675–677.

Pavše, L., Tul, N., & Velikonja, V. (2019). Analiza notranje strukture slovenskega prevoda Lestvice vezi med nosečnico in plodom (PAI) [Analysis of the internal structure of the Slovenian version of the Prenatal Attachment Inventory (PAI)]. *Psihološka obzorja*, *28*, 11–18.

Yalçın, S. S., Örün, E., Özdemir, P., Mutlu, B., & Dursun, A. (2014). Türk annelerde doğum sonrası bağlanma ölçeklerinin güvenilirliği. [Reliability of postnatal attachment scales in Turkish mothers]. *Cocuk Sagligi ve Hastaliklari Dergisi, 57 (4),* 246–251.

## Appendix C Reference list of included studies (*n* = 65)

Andrek, A., Kekecs, Z., Hadhazi, E., & Boukydis, Z. & Varga, K. (2016). Re-Evaluation of the psychometric properties of the Maternal-Fetal Attachment Scale in a Hungarian sample. *Journal of Obstetrics, Gynaecology and Neonatal Nursing, 45(5)*, e15–25.

Baldisserotto, M. L., Theme-Filha, M. M., Harter Griep, R. Oates, J., Reno Junior, J., & Pires Cavalsan, J. (2018). Transcultural adaptation to the Brazilian Portuguese of the Postpartum Bonding Questionnaire for assessing the postpartum bond between mother and baby. *Cadernos de Saude Publica, 34(7)*: e00170717.

Barone, L., Lionetti, F., & Dellagiulia, A. (2014). Maternal-fetal attachment and its correlates in a sample of Italian women: a study using the Prenatal Attachment Inventory. *Journal of Reproductive and Infant Psychology, 32(3),* 230–239.

Bhakoo, O. N., Pershad, D., Mahajan, R., & Gambhir, S. K. (1994). Development of mother-infant attachment scale. *Indian Pediatrics, 31*, 1477–1482.

Bienfait, M., Maury, M., Haquet, A., Faillie, J.L., Franc, N., … Cambonie, G. (2011). Pertinence of the self-report mother-to-infant bonding scale in the neonatal unit of a maternity ward. *Early Human Development, 87(4)*, 281–287.

Brockington, I. F., Fraser, C. & Wilson, D. (2006). The Postpartum Bonding Questionnaire: a validation. *Archives of Women's Mental Health, 9*, 233–242.

Brockington, I. F., Oates, J., George, S., Turner, D., Vostanis, P., Sullivan, M., Loh, C. & Murdoch, C. (2001). A Screening Questionnaire for mother-infant bonding disorders. *Archives of Women's Mental Health, 3*, 133–140.

Busonera, A., Cataudella, S., Lampis, J., Tommasi, M. & Zavattini, G. C. (2016a). Psychometric properties of a 20-item version of the Maternal-Fetal Attachment Scale in a sample of Italian expectant women. *Midwifery, 34*, 79–87.

Busonera, A., Cataudella, S., Lampis, J., Tommasi, M. & Zavattini, G. C. (2016b). Investigating validity and reliability evidence for the maternal antenatal attachment scale in a sample of Italian women. *Archives of Women's Mental Health, 19*, 329–336.

Busonera, A., Cataudella, S., Lampis, J., Tommasi, M., & Zavattini, G. C. (2017a). Prenatal Attachment Inventory: expanding the reliability and validity evidence using a sample of Italian women. *Journal of Reproductive and Infant Psychology, 35(5)*, 462–479.

Busonera, A., Cataudella, S., Lampis, J., Tommasi, M., & Zavattini, G. C. (2017b). Psychometric properties of the Postpartum Bonding Questionnaire and correlates of mother-infant bonding impairment in Italian new mothers. *Midwifery, 55*, 15–22.

Chen, C.-J., Sung, H.-C., Chen, Y.-C., Chang, C.-Y., & Lee, M.-S. (2013). The development and psychometric evaluation of the Chinese version of the maternal attachment inventory. *Journal of Clinical Nursing, 22*(19–20), 2685– 2695.

Condon, J. T. (1993). The assessment of antenatal emotional attachment: Development of a questionnaire instrument. *British Journal of Medical Psychology, 66*, 167–183.

Condon, J. T., & Corkindale, C. (1997). The correlates of antenatal attachment in pregnant women. *Psychology and Psychotherapy: Theory, Research and Practice*, *70*(4), 359–372.

Condon, J. T., & Corkindale, C. J. (1998). The assessment of parent-to-infant attachment: Development of a self-report questionnaire instrument. *Journal of Reproductive and Infant Psychology*, *16*(1), 57–76.

Condon, J. T., Corkindale, C. J. & Boyce, P. (2008). Assessment of postnatal paternal–infant attachment: development of a questionnaire instrument. *Journal of Reproductive and Infant Psychology, 26(3)*, 195–210.

Condon, J. T., Corkindale, C., Boyce, P. & Gamble, E. (2013). A longitudinal study of father-to-infant attachment: Antecedents and correlates. *Journal of Reproductive and Infant Psychology, 31(1)*, 15–30.

Cranley, M. S. (1981). Development of a tool for the measurement of maternal attachment in pregnancy. *Nursing Research, 30(5)*, 281–284.

Cuijlits, I., van de Wetering, A. P., Potharst, E. S., Truijens, S. E. M., van Baar, A. L., & Pop, V. J. M. (2016). Development of a Pre- and Postnaal Bonding Scale (PPBS). *Journal of Psychology & Psychotherapy, 6(5)*: 1000282.

Della Vedova, A. M., & Burrp, R. (2017). Surveying prenatal attachment in fathers: the Italian adaptation of the Paternal Antenatal Attachment Scale (PAAS-IT). *Journal of Reproductive and Infant Psychology, 35(5)*, 493–508.

Della Vedova, A. M., Dabrassi, F., & Imbasciati, A. (2008). Assessing prenatal attachment in a sample of Italian women. *Journal of Reproductive and Infant Psychology, 26(2)*, 86–98.

Doster, A., Wallwiener, S., Müller, M., Matthies, L. M., Plewniok, K., … Reck, C. (2018). Reliability and validity of the German version of the Maternal-Fetal Attachment Scale. *Archives of Gynecology and Obstetrics, 297*, 1157–1167.

Ekström, A., & Nissen, E. (2006). A mother’s feelings for her infant are strengthened by excellent breastfeeding counseling and continuity of care. *Pediatrics, 118(2)*, e309–314.

Feldstein, S., Hane, A. A., Morisson, B. M., Huang, K-Y. (2004). Relation of the Postnatal Attachment Questionnaire to the Attachment Q-Set. *Journal of Reproductive and Infant Psychology, 22(2)*, 111–121.

Garcia-Esteve, L., Torres, A., Lasheras, G., Palacios-Hernández, B., Farré-Sender, B., Subirà, S., Valdés, M. & Brockington, I.F. (2016). Assessment of psychometric properties of the Postpartum Bonding Questionnaire (PBQ) in Spanish mothers. *Archives of Women's Mental Health, 19(2)*, 385–394.

Gau, M.-L., & Lee, T-Y. (2003). Construct validity of the Prenatal Attachment Inventory: A confirmatory factor analysis approach. Journal of Nursing Research, *11(3)*, 177–186.

Golbasi, Z., Ucar, T., & Tugut, N. (2015). Validity and reliability of the Turkish version of the Maternal Antenatal Attachment Scale. *Japan Journal of Nursing Science, 12*, 154–161.

Göbel, A., Barkmann, C., Goletzke, J., Hecher, K., Schulte-Markwort, M., … Mudra, S. (2019). Psychometric properties of 13-item versions of the maternal and paternal antenatal attachment scales in German. *Journal of Reproductive and Infant Psychology,* doi: 10.1080/02646838.2019.1643833.

Hackney, M., Braithwaite, S., Radcliff, G. (1996). Postnatal depression: the development of a self- report scale. *Health Visitor, 169*, 103–104.

Hoivik, M. S., Burkeland, N. A., Linaker, O. M., & Berg-Nielsen, T. S. (2013). The Mother and Baby Interaction Scale: a valid broadband instrument for efficient screening of postpartum interaction? A preliminary validation in a Norwegian community sample. *Scandinavian Journal of Caring Sciences, 27*, 733–739.

Kaneko, H., & Honjo, S. (2014). The psychometric properties and factor structure of the Postpartum Bonding Questionnaire in Japanese mothers. *Psychology*, *5(9)*, 1135.

Kinsey, C. B., Baptiste-Roberts, K., Zhu, J., & Kjerulff, K. H. (2014). Birth-related, psychosocial, and emotional correlates of positive maternal-infant bonding in a cohort of first-time mothers. *Midwifery, 30*, e188–e194.

Leifer, M. (1997). Psychological changes accompanying pregnancy and Motherhood. *Genetic Psychology Monographs, 95(1)*, 55–96.

Lingeswaran, A., & Bindu, H. (2012). Validation of Tamil Version of Cranley’s 24-Item Maternal–Fetal Attachment Scale in Indian Pregnant Women. *The Journal of Obstetrics and Gynecology of India*, *62(6)*, 630–634.

Mako, H. S., & Deak, A. (2014). Reliability and validity of the Hungarian version of the Maternal Antenatal Attachment Scale. *International Journal of Gynecological and Obstetrical Research, 1*, 1–13.

Müller, M. E. (1993). Development of the Prenatal Attachment Inventory. *Western Journal of Nursing Research, 15(2)*, 199–215.

Müller, M. E. (1994). A questionnaire to measure mother-to-infant attachment. *Journal of Nursing Measurement, 2(2)*, 129–141.

Müller, M. E. (1996). Prenatal and postnatal attachment: A modest correlation. *Journal of Obstetrics, Gynaecology and Neonatal Nursing, 25(2)*, 161–166.

Müller, M. E., & Ferketich, S. (1993). Factor analysis of the Maternal Fetal Attachment Scale. *Nursing Research, 42(3)*, 144–147.

Navarro-Aresti, L., Iraurgi, I., Iriarte, L., & Martinez-Pampliega, A. (2016). Maternal Antenatal Attachment Scale (MAAS): Adaptation to Spanish and proposal for a brief version of 12 items. *Archives of Women's Mental Health, 19,* 95–103.

Noh, N. I., & Yeom, J.-A. (2017). Development of the Korean Paternal-Fetal Attachment Scale (K-PAFAS). *Asian Nursing Research, 11*, 98–106.

Oates, J. and Gervai, J. (2019). Mothers’ perceptions of their infants, *Journal of Prenatal and Perinatal Psychology and Health* (in press).

Oates, J., Gervai, J., Danis, I., Lakatos, K., & Davies, J. (2018). Validation of the Mothers’ Object Relations Scales Short-form (MORS-SF). *Journal of Prenatal and Perinatal Psychology and Health, 33(1)*, 38–50.

Ohara, M., Okada, T., Kubota, C., Nakamura, Y., Shiino, T., Aleksic, B., Morikawa, M., Yamauchi, A., Uno, Y., Murase, S. & Goto, S., (2016). Validation and factor analysis of mother-infant bonding questionnaire in pregnant and postpartum women in Japan. *BMC Psychiatry, 16*: 212.

Ohashi, Y., Kitamura, T., Sakanashi, K., & Tanaka, T. (2016). Postpartum bonding disorder: factor structure, validity, reliability and a model comparison of the Postnatal Bonding Questionnaire in Japanese mothers of infants. *Healthcare, 4(50)*, 1–11.

Omani Samani, R., Maroufizadeh, S., Ezabadi, Z., Alizadeh, L., & Vesali, S. (2016). Psychometric properties of the Persian version of the Prenatal Attachment Inventory in pregnant Iranian women. *International Journal of Fertility and Sterility, 10(2)*, 184–189.

Pallant, J. F., Haines, H. M., Hildingsson, I., Cross, M. & Rubertsson, C. (2014). Psychometric evaluation and refinement of the Prenatal Attachment Inventory. *Journal of Reproductive and Infant Psychology, 32(2)*, 112–125.

Reck, C., Klier, C.M., Pabst, K., Stehle, E., Steffenelli, U., Struben, K. & Backenstrass, M. (2006). The German version of the Postpartum Bonding Instrument: psychometric properties and association with postpartum depression. *Archives of Women's Mental Health, 9(5)*, 265–271.

Riera-Martin, A., Oliver-Roig, A., Martinez-Pampliega, A., Cormenzana-Redondo, S., Clement-Carbonell, & Richart-Martinez, M. (2018). A single Spanish version of maternal and paternal postnatal attachment scales: validation and conceptual analysis. *PeerJ, 6*, e5980.

Scopesi, A., Viterbori, P., Sponza, S., & Zucchinetti, P. (2004). Assessing mother-to-infant attachment: the Italian adaptation of a self-report questionnaire. *Journal of Reproductive and Infant Psychology, 22(2)*, 99–109.

Seimyr, L., Sjögren, B., Welles-Nystrom, B. & Nissen, E. (2009). Antenatal maternal depressive mood and parental-fetal attachment at the end of pregnancy. *Archives of Women's Mental Health, 12(5)*, 269–279.

Shin, H., & Kim, Y. H. (2007). Maternal Attachment Inventory: Psychometric evaluation of the Korean version. *Journal of Advanced Nursing, 59(3)*, 229–307.

Siu, B.W.M., Ip, P., Chow, H.M.T., Kwok, S.S.P., Li, O.L., Koo, M.L., Cheung, E.F.C., Yeung, T.M.H. and Hung, S.F., (2010). Impairment of mother-infant relationship: validation of the Chinese version of Postpartum Bonding Questionnaire. *Journal of Nervous and Mental Disease, 198(3)*, 174–179.

Sjögren, B., Edman, G., Widstrom, A. M., Mathieson, A. S., & Uvnas-Moberg, K. (2004). Maternal foetal attachment and personality during first pregnancy. *Journal of Reproductive and Infant Psychology, 22(2)*, 57–69.

Suetsugu, Y., Honjo, S., Ikeda, M. & Kamibeppu, K. (2015). The Japanese version of the Postpartum Bonding Questionnaire: Examination of the reliability, validity, and scale structure. *Journal of Psychosomatic Research, 79(1)*, 55–61.

Taylor, A., Atkins, R., Kumar, R., Adams, D. & Glover, V. (2005). A new Mother-to-Infant Bonding Scale: links with early maternal mood. *Archives of Women's Mental Health, 8(1)*, 45–51.

Thorstensson, S., Hertfelt Wahn, E., Ekström, A., & Langius-Eklöf, A. (2012). Evaluation of the Mother-to-Infant relation and feeling scale: Interviews with first-time mothers for feelings and relation to their baby three days after birth. *International Journal of Nursing and Midwifery, 4(1)*, 8–15.

Thorstensson, S., Nissen, E., & Ekstrom, A. (2012). Professional support in pregnancy influence maternal relation to and feelings for the baby after cesarean birth: an intervention study. *Journal of Nursing & Care, 1(4)*, 1–9.

van Bussel, J. C. H., Spitz, B. & Demyttenaere, K. (2010a). Three self-report questionnaires of the early mother-to-infant bond: reliability and validity of the Dutch version of the MPAS, PBQ and MIBS. *Archives of Women's Mental Health, 13,* 373–384.

van Bussel, J. C. H., Spitz, B. & Demyttenaere, K. (2010b). Reliability and validity of the Dutch version of the maternal antenatal attachment scale. *Archives of Women's Mental Health, 13,* 267–277.

Vengadavaradan, A., Bharadwaj, B., Sathynarayanan, G., Durairaj, J., & Rajaa, S. (2019). Translation, validation and factor structure of the Tamil version of the Postpartum Bonding Questionnaire (PBQ-T). *Asian Journal of Psychiatry, 40*, 62–67.

Weaver, R. H., & Cranley, M. S. (1983). An exploration of paternal-fetal attachment behavior. *Nursing Research, 32*(2), 68–72.

Wittkowski, A., Wieck, S. & Mann, S. (2007). An evaluation of two bonding questionnaires: a comparison of the Mother-to-Infant Bonding Scale with the Postpartum Bonding Questionnaire in a sample of primiparous mothers. *Archives of Women's Mental Health, 10(4)*, 171–175.

Wittkowski, A., Williams, J. & Wieck, A. (2010). An examination of the psychometric properties and factor structure of the Post-partum Bonding Questionnaire in a clinical inpatient sample. *British Journal of Clinical Psychology, 49(2)*, 163–172.

Yoshida K., Y., H., Conroy, S., Marks, M. & Kumar, C. (2012). A Japanese version of Mother-to-Infant Bonding Scale: factor structure, longitudinal changes and links with maternal mood during the early postnatal period in Japanese mothers. *Archives of Women's Mental Health, 15(5)*, 343–352.
